# *Enterococcus faecalis* Infection Causes Inflammation, Intracellular Oxphos-Independent ROS Production, and DNA Damage in Human Gastric Cancer Cells

**DOI:** 10.1371/journal.pone.0063147

**Published:** 2013-04-30

**Authors:** Jesper A. B. Strickertsson, Claus Desler, Tomas Martin-Bertelsen, Ana Manuel Dantas Machado, Torkel Wadstrøm, Ole Winther, Lene Juel Rasmussen, Lennart Friis-Hansen

**Affiliations:** 1 Center for Genomic Medicine, Rigshospitalet, University of Copenhagen, Copenhagen, Denmark; 2 Department of Veterinary Clinical and Animal Sciences, Faculty of Health and Medical Sciences, University of Copenhagen, Copenhagen, Denmark; 3 Center for Healthy Ageing, Faculty of Health and Medical Sciences, University of Copenhagen, Copenhagen, Denmark; 4 Department of Biology and Biotech Research and Innovation Centre, The Bioinformatics Centre, Faculty of Science, University of Copenhagen, Copenhagen, Denmark; 5 Department of Clinical Microbiology, University of Lund, Lund, Sweden; 6 DTU Informatics, Technical University of Denmark, Copenhagen, Denmark; National Cancer Institute, United States of America

## Abstract

**Background:**

Achlorhydria caused by e.g. atrophic gastritis allows for bacterial overgrowth, which induces chronic inflammation and damage to the mucosal cells of infected individuals driving gastric malignancies and cancer. *Enterococcus faecalis* (*E. faecalis*) can colonize achlohydric stomachs and we therefore wanted to study the impact of *E. faecalis* infection on inflammatory response, reactive oxygen species (ROS) formation, mitochondrial respiration, and mitochondrial genetic stability in gastric mucosal cells.

**Methods:**

To separate the changes induced by bacteria from those of the inflammatory cells we established an *in vitro E. faecalis* infection model system using the gastric carcinoma cell line MKN74. Total ROS and superoxide was measured by fluorescence microscopy. Cellular oxygen consumption was characterized non-invasively using XF24 microplate based respirometry. Gene expression was examined by microarray, and response pathways were identified by Gene Set Analysis (GSA). Selected gene transcripts were verified by quantitative real-time polymerase chain reaction (qRT-PCR). Mitochondrial mutations were determined by sequencing.

**Results:**

Infection of MKN74 cells with *E. faecalis* induced intracellular ROS production through a pathway independent of oxidative phosphorylation (oxphos). Furthermore, *E. faecalis* infection induced mitochondrial DNA instability. Following infection, genes coding for inflammatory response proteins were transcriptionally up-regulated while DNA damage repair and cell cycle control genes were down-regulated. Cell growth slowed down when infected with viable *E. faecalis* and responded in a dose dependent manner to *E. faecalis* lysate.

**Conclusions:**

Infection by *E. faecalis* induced an oxphos-independent intracellular ROS response and damaged the mitochondrial genome in gastric cell culture. Finally the bacteria induced an NF-κB inflammatory response as well as impaired DNA damage response and cell cycle control gene expression.

**Transcript profiling:**

Array Express accession number E-MEXP-3496.

## Introduction

Gastric cancer is among the ten most common cancers, and with a global annual death rate of approximately 700.000, it is the second most common cause of cancer related mortality [1]. The intestinal type gastric cancer develops through a series of pathological events starting with chronic inflammation, atrophic gastritis, intestinal metaplasia, and finally cancer [2].

Chronic inflammation and cancer has been linked in several studies of patients and of genetically modified mice, and is believed to be involved in the pathogenesis of about 25% of all cancer cases worldwide [2]–[4]. Characteristics of cancer-related inflammation include the presence of chemokines and cytokines in tumor tissues, having the potential to stimulate tumor-cell proliferation and survival of malignant cells [5], [6]. Chronic inflammation also favors an overproduction of DNA damaging reactive oxygen species (ROS), whose production can be incidental to oxidative phosphorylation (oxphos) reactions in the mitochondria (oxphos-dependant) or produced from outside the mitochondria most commonly by nicotinamid adenine dinucleotide phosphate (NADPH) oxidases (oxphos-independent) (for review see [7]–[9]). Chronic production of ROS cause DNA damage, allowing the accumulation of mutations which in turn can activate oncogenes and/or inactivate tumor suppressor genes thereby increasing the risk of cancer development [3].

The most common risk factor for developing gastric cancer is chronic bacterial infection of the stomach with *Helicobacter pylori* (*H. pylori*) [10]. Chronic infection of the stomach by *H. pylori* affect the gastric pH balance and can cause achlorhydria or hyperchlorhydria [11]. Although this bacterium is classified as a class one carcinogen, it is not always associated with an increased risk of gastric cancer development. For instance, *H. pylori* infected patients with duodenal ulcers and high levels of gastric acid have a reduced risk of developing gastric cancer in comparison to those from the general population [11]–[13]. In contrast, patients with atrophic gastritis and reduced gastric acid secretion have an increased risk of developing gastric cancer [11], [13], [14]. The increased cancer risk in achlorhydric individuals could be due to bacterial overgrowth of other bacteria in the gastric lumen [15]. In both achlorhydric humans and animal models bacterial overgrowth cause chronic gastritis which develops into intestinal metaplasia and finally gastric cancer [16]–[18]. Among the bacteria found in the stomach of achlorhydric mice were *Enterococci* species, which are gram-positive cocci able to survive in environments with a pH as low as 4.5 [19], [20]. *Enterococcus faecalis* (*E. faecalis*) is a member of the human commensal microbiota and one of the most common bacteria in the gastrointestinal tract [19]. In spite of this, *E. faecalis* can act as a human pathogen [21], and has been found in significantly increased numbers in oral cancerous lesions and in human colon cancers [22], [23]. In relation to this *E. faecalis* is capable of producing N-nitrosamines and of inducing genetic instability in colonic epithelial cells through oxidative damage of the DNA [24], [25].

Gastritis is associated with infiltration of immune cells in the tissue, which makes it difficult to dissect the action of the immune cells from that of the lining mucosal cells *in vivo*. We therefore used an *in vitro* tissue culture model that allowed us to examine how isolated mucosal cells respond to bacteria and the molecular mechanisms by which intestinal bacteria such as *E. faecalis* induce damage in gastric epithelial cells. Using this model we examined the impact of *E. faecalis* infection of gastric adenocarcinoma cell cultures on ROS production, cellular respiration, growth, DNA damage/repair and inflammatory responses.

## Materials and Methods

### Cell Culture, *E. faecalis*, and Growth Conditions

Human MKN74 gastric adenocarcinoma cell cultures from the Japanese Collection of Research Bioresources cell bank (JCRB #JCRB0255) were maintained in RPMI 1640 medium (Invitrogen, Carlsbad, CA), supplemented with 10% fetal bovine serum (FBS) (Invitrogen), 100 µg/ml streptomycin, and 100 U/ml penicillin (Invitrogen) at 37°C, and 5% CO_2_ humidified atmosphere. Infections were performed with *E. faecalis* strain (ATCC 29212). Bacteria were grown in 5% blood agar plates at 37°C. Optical density (OD) of bacteria grown in RPMI 1640 medium was measured at 550 nm on a UV-1601 spectrophotometer (Shimadzu, Kyoto, Japan) (Figure S1). *E. faecalis* lysate was prepared by freeze/thawing the bacterial suspension three times, while sonicating the suspension between each cycle.

### Infection of Gastric Cells for RNA and DNA isolation

For 24 h infections, 80% confluent MKN74 cells were washed with PBS and incubated in antibiotic free medium. Overnight-grown colonies of *E. faecalis* were added to the MKN74 cell culture at a multiplicity of infection (MOI) of 50 bacteria per cell. 5 day infections were carried out by treating 65% confluent MKN74 cells with *E. faecalis* at a MOI of 10 or with a lysate protein concentration of 40 µg/µl. Every 24 h, cells were washed with PBS and fresh medium and bacteria or lysate were added. Control cells were processed similarly in the absence of bacteria or bacterial lysate. *In vitro* studies were performed using a gastric cancer cell line to test the damaging effect of *E. faecalis* on gastric adenocarcinoma cells. These cells differ from normal gastric epithelial cells by carrying several chromosomal aberrations, but offer the advantage of being a reproducible model system that in many ways replicates the events occurring in the stomach. Comparing infected cells to uninfected cells gives a reliable picture of damage and alterations in gene expression caused by the infection.

### RNA and DNA isolation

After infection, RNA and DNA were extracted by adding Trizol Reagent (Invitrogen) to each culture flask. RNA was isolated according to the manufactures protocol. The DNA-containing intermediate phase was precipitated by adding 100% ethanol. Samples were centrifuged at 4°C, 3500 g for 6 min. The phenol-ethanol supernatant was removed and the remaining DNA extraction was done by using a NucleoSpin tissue kit (Macherey-Nagel, Düren, Germany). RNA and DNA concentrations were measured on a NanoDrop ND-1000 Spectrophotometer. RNA integrity numbers were measured on a 2100 Bioanalyzer (Agilent Technologies, Santa Clara, California) according to the manufactures protocol.

### Measurement of ROS and Superoxide

Two days prior to infection 8×10^4^ MKN74 cells were plated in each well of a Lab-Tek^TM^ Chambered Coverglas (Thermo Fisher Scientific, Waltham, Massachusetts). MKN74 cells were stained for two hours prior to infection with 2-plex detection mix. ROS and superoxide staining was performed using the ROS/RNS Detection Kit (Enzo Life Sciences, Farmingdale, New York) according to the manufactures instructions. MKN74 cells were infected at MOI of 50 for 30 min and analyzed under a Zeiss LSM 510 confocal microscope using the LSM 510 software (Zeiss, Oberkochen, Germany). Uninfected stained MKN74 cells were used as negative controls.

### Localization of *E. faecalis* during infection

8×10^4^ MKN74 cells/well were seeded in an 8-chamber glass-bottom slide (Thermo Fisher Scientific) 24 hours prior to staining. In order to identify the localization of *E. faecalis* after infection we separately stained the plasma membrane with CellMask^TM^ Deep Red plasma membrane stain (C10046, Invitrogen) and the bacterial cell wall using BacLight^TM^ Green bacterial stain (B-35000, Molecular probes, Invitrogen) according to the two protocols respectively. The cells and bacteria were washed 3 times in PBS prior to infection. The cells were infected at MOI of 100 for 4 hours and analyzed under a Zeiss LSK 510 confocal microscope using the LSM 510 software (Zeiss). This experiment was repeated three times and 8 champers were examined each time.

### XF24 Microplate based respirometry

Respirometry of MKN74 cells was performed using an XF24 Extracellular Flux Analyzer (Seahorse Bioscience, North Billerica, MA). Half of the plated MKN74 cells were infected with *E. faecalis* at a MOI of 50 for 4, 8 or 24 hours while the other half was left uninfected. After incubation, bacteria were removed using 4% Penicillin/streptomycin (5 U/ml) and 4% cefotaxim (100 µg/ml) (ACS Dobfar Generics S.A., Luxembourg, Belgium). Cells were incubated in a CO_2_ free incubator at 37°C for 1 hour to allow temperature and pH equilibration. The microplate with cells was then loaded into the XF24 and the oxygen consumption rate of each well was measured over a period of 100 minutes. The drugs oligomycin (0.5 µM), FCCP (0.3 µM) and antimycin A (2.0 µM) were in turn added to each well. More details are available in File S1.

### Mitochondrial DNA Instability

The frequency of mutations in the D-loop region of mitochondrial DNA, were determined by PCR amplification using primers C6-CA5 (Table S1). The amplified fragments were cloned into the pCR2.1 vector (Invitrogen) and inserts from 328 colonies were amplified using the VectorD-loop primers (Table S1). The amplified fragments were purified and sequenced using the ABI Prism BigDye Terminator Cycle Sequencing Kit and an ABI Prism 3730 DNA Sequencer (Perkin-Elmer, Waltham, Massachusetts). To determine mutations, the sequences were used as queries against the Cambridge reference sequence obtained from MitoMap (http://www.mitomap.org. Accessed Feb 9. 2012).

### Microarray and GSA

RNA with a RNA integrity number of 8 or above from three control and three infection samples (with viable *E. faecalis* or with *E. faecalis* lysate 40 µg/µl) for 24 hour and 5 day experiments were submitted to the RH Microarray Center at the Copenhagen University Hospital. RNA was amplified and labeled using the 3′ IVT Express kit (Affymetrix, Santa Clara, CA, USA) according to manufactures instructions. 250 ng total RNA was used as input. The labeled samples were hybridized to GeneChip Genome U133 plus 2 arrays (Affymetrix). The arrays were washed and stained with phycoerytrin conjugated streptavidin using a Affymetrix Fluidics Station® 450, and the arrays were scanned in a Affymetrix GeneArray® 2500 scanner to generate fluorescent images, as described in the Affymetrix GeneChip® protocol. 24 raw cell intensity files (CEL-files) were generated in the GeneChip® Command Console® Software (AGCC) (Affymetrix). The raw CEL-files were made publicly available at ArrayExpress with accession number E-MEXP-3496. Preprocessing of microarrays for the gene set analysis (GSA) was done with R/Bioconductor [26], [27]. Background adjustment, quantile normalization and final summarization to probe set expression intensities was performed by the GCRMA algorithm [28] for each experimental condition, separately (File S2).

### Quantitative Reverse transcription PCR

cDNA was synthesized using SuperScript III First-Strand Synthesis SuperMix for qPCR (Invitrogen). Gene expression was analyzed by qRT-PCR using SYBR GreenER q-PCR SuperMix for ABI PRISM (Invitrogen). Primers were designed using the Primer-Blast software from NCBI [29]. The reactions were run on an ABI PRISM 7900HT Sequence Detection System (SDS) from Applied Biosystems. Data was normalized to GAPDH mRNA expression.

### Growth assay

8000 cells were distributed to each well of six 24-well plates and incubated at 37°C and 5% CO_2_ for two days prior to treatment. Cells were treated in quadruplicates as follows; 4 wells of uninfected control cells, 4 wells treated with 400 µg/μl *E. faecalis* lysate, 4 wells treated with 40µg/μl lysate, 4 wells treated with 4 µg/μl lysate and 4 wells treated with viable *E. faecalis* at a MOI of 10. One plate of cells was fixated every day. Fixation was done by washing with PBS and fixating with 1 ml 10% formalin for 10 min. Fixated cells were stained with 1 ml of 0.1% crystal violet (Sigma-Aldrich, St. Louis, Missouri), and plates were shaken for 30 min. Cells were washed, dried, and crystal violet was extracted with 500 µl 10% acetic acid. Absorbance was measured at 620 nm on a PowerWave XS (BioTek Instruments, Winooski, Vermont).

### Statistical Analysis

Single classification analysis of variance (ANOVA) was used to test differences in maximal respiratory capacity, ATP turnover and oxphos-independent oxygen consumption. ANOVA was furthermore used to test differences in cell growth during different treatments with *E. faecalis*. When the ANOVA indicated significant differences among the treatments, Tukeys honestly significant method was used to test for differences between oxygen consumption rates or number of cells. mtDNA mutation frequencies were assessed by chi-square and Fisher's exact test. ROS results were expressed as mean values of at least twenty independent measurements and analyzed by unpaired two-tailed *t*-test. qRT-PCR results were expressed as mean values of at least three independent experiments measured in three technical replicates, and analyzed by unpaired two-tailed *t*-test. The differences between data sets were considered significant at p values ≤0.05. Error bars  =  ± SEM unless otherwise indicated.

Identification of regulated pathways was done by a GSA method, GAGE [30], enhanced to exploit available induction-repression information (for extensive details and calculations see File S2). Pathways were represented by lists of genes (gene sets) downloaded from the Molecular Signatures Database (MSigDB) 3.0 [31]. C2 collection of curated gene sets (canonical pathways and gene expression signatures of genetic and chemical perturbations) using gene symbols identifiers. Gene set p-values were corrected for multiple testing by estimating the false discovery rate and significance level was set at 1%.

## Results

### *E. faecalis* infection induce intracellular ROS and superoxide production

**Figure 1:**
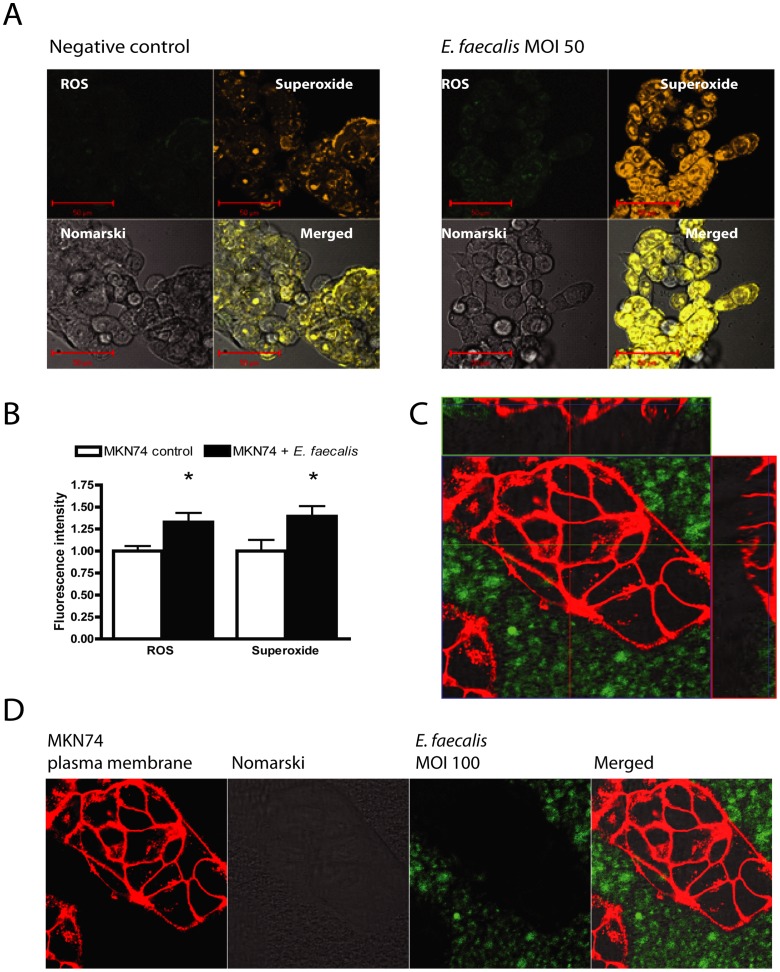
*E. faecalis* infection induced intracellular ROS production. MKN74 cells infected with *E. faecalis* for 30 minutes at MOI50. (A) Representative fluorescence microscope image of MKN74 cells stained with ROS detecting probes (green) and superoxide detecting probes (orange) Scale bars  = 50 µm. (B) Quantification of fluorescence intensity using the LSM 510 software. A statistically significant increased intracellular production of ROS (p<0.01) and superoxide (p<0.02) in the infected cells compared to uninfected control cells was observed. * denotes significant difference. (C) Fluorescence image showing MKN74 plasma membrane (red) and *E. faecalis* (green) after 4 hours of infection. Flanking images show the YZ and XZ plane. (D) Split image of C. No evidence for bacterial invasion of the cells was observed.

Others and we have previously shown that the achlorhydric gastrin KO mouse had bacterial overgrowth with *enterococci* that are normally not part of the gastric flora [20]. Although *Enterococci* in general are thought to be of low pathogenicity, sustained overgrowth in achlorhydric mice is associated with an inflammatory response and ultimately these mice develop gastric cancer [20], [32]. To characterize the pathological process within the gastric cells we examined how the bacteria affected the gastric epithelial cells. Using probes monitoring intracellular ROS production, we found that 30 minutes of infection stimulated a significant increase in intracellular ROS formation (Figure 1A). With probes specific for superoxide production we showed that the ROS mainly consisted of superoxide in MKN74 cells (Figure 1A). Fluorescence intensity was quantified using the LSM 510 software and a statistically significant increase in intracellular ROS and superoxide in the infected cells compared to uninfected control cells was observed (Figure 1B). We found no evidence that suggested invasion of the cells by bacteria so we conclude that the intracellular ROS was produced by the infected cells and not by internalized *E. faecalis* (Figure 1 C–D).

### Infection decreases mitochondrial activity and increases oxphos-independent oxygen consumption

**Figure 2:**
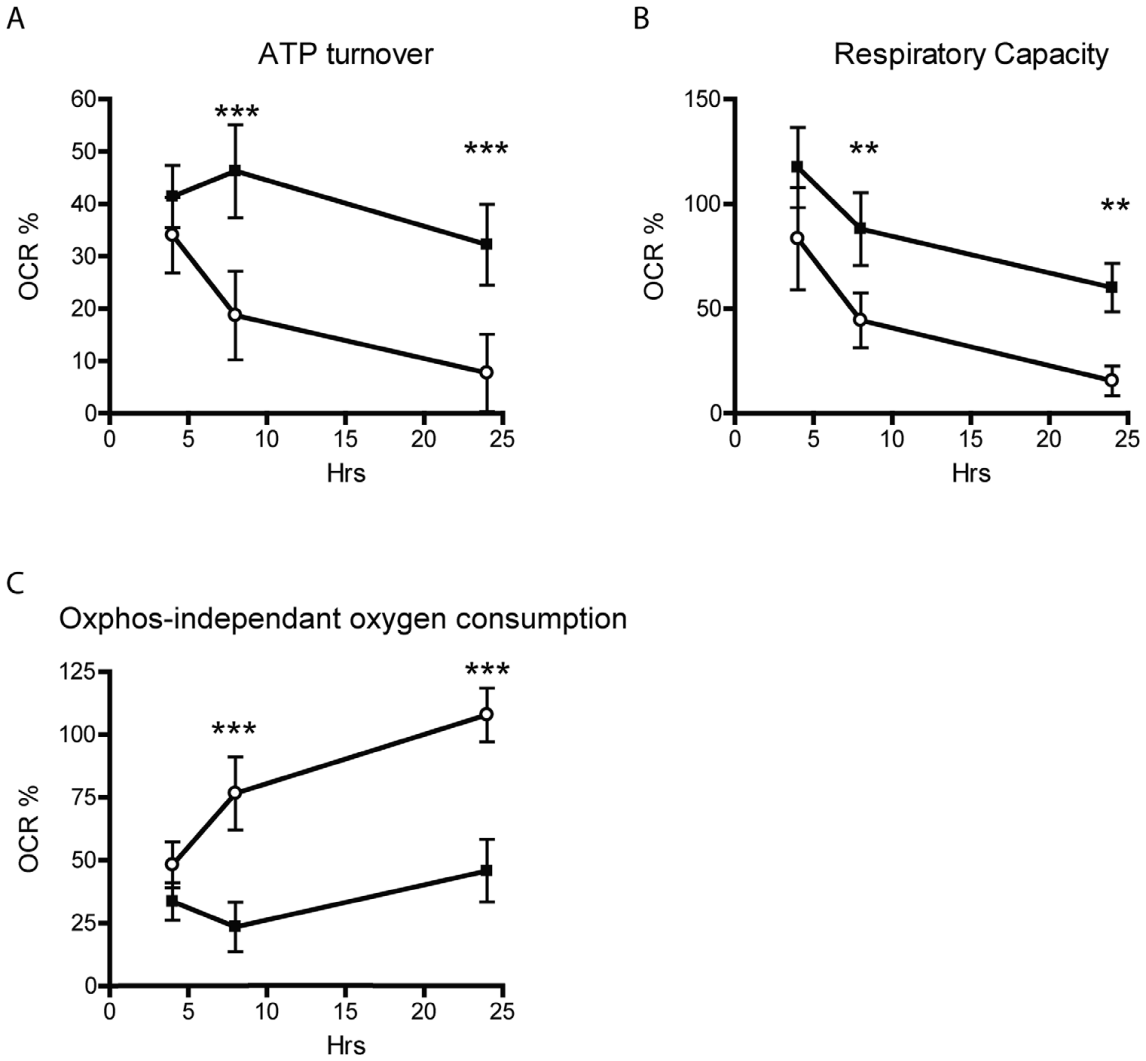
Intracellular oxygen consumption in MKN74 cells was independent of oxidative phosphorylation after infection. MKN74 cells were incubated with or without *E. faecalis* for 4, 8 or 24 hours. Bacteria were removed and oxygen consumption rate was measured in an XF24 Extracellular Flux Analyzer. (A) ATP turnover, (B) respiratory capacity and (C) oxphos-independant oxygen consumption was determined as described in the text. **▪** =  Control cells, **ο**  =  *E. faecalis* infected cells. (n = 3–6, error bars indicate S.D. ** and *** denotes significant difference p<0.01 and p<0.001 respectively).

The source of ROS found following bacterial infection could either be generated by extracellular bacteria [33] and/or produced from within the cells [34], [35]. Intracellular ROS can either be generated in an oxphos-dependent or independent manner. To examine and subsequently characterize the possible intracellular ROS production associated with *E. faecalis* infection; we measured how the bacterial infection affected mitochondrial respiration by measuring ATP turnover, respiratory capacity and oxphos-independent oxygen consumption (Figure 2). To insure that the results reflected respiration of the MKN74 cells only, all bacteria were removed prior to the measurements. There was no significant difference in ATP turnover of control cells grown for 4–24 hours, whereas a significant 2- and 4 fold decrease (p<0.001) of ATP turnover were present between control cells and infected cells incubated for 8 and 24 hours respectively (Figure 2A). A corresponding correlation was found in the respiratory capacity, where the capacities of infected cells were also significantly 2- and 4 fold decreased (p<0.01) compared to control cells after 8 and 24 hours of infection (Figure 2B). The oxphos-independent oxygen consumption of control cells was unaltered for cells grown for 4–24 hours. In contrast the oxphos-independent oxygen consumption of cells infected with bacteria increased from ∼50% of total oxygen uptake after 4 hours of infection to become the primary oxygen utilizer (p<0.001) (Figure 2C). This suggests that *E. faecalis* interact with the epithelial cells inducing ROS formation and oxygen consumption by other intracellular enzyme systems than oxidative phosphorylation. Expression of NOX1-5 and Duox1-2 was examined in the gastric cell line; however, expression was not affected by the infection (Table S2).

### *E. faecalis* infection caused mitochondrial DNA instability

**Figure 3:**
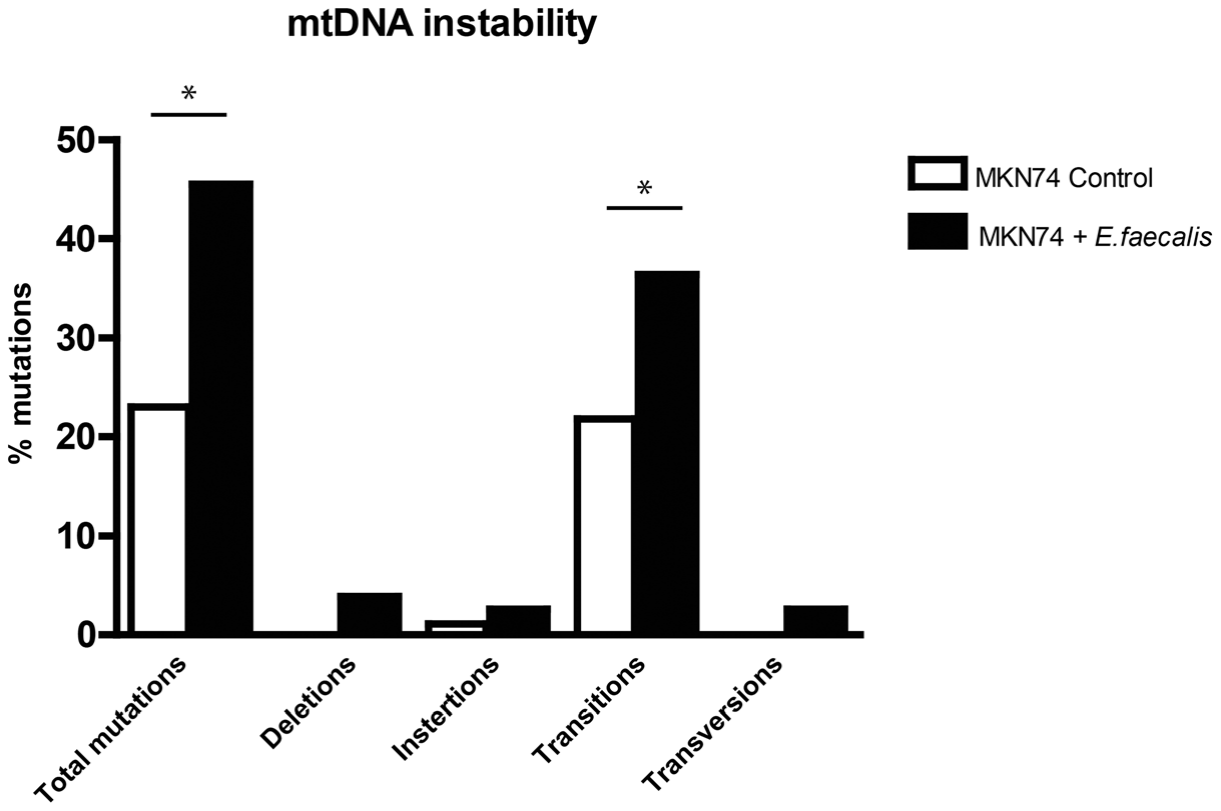
*E. faecalis* infection increased mitochondrial mutations in MKN74 cells. Mutations detected in the mtDNA D-loop region after infection. MKN74 cells were infected with *E. faecalis* at MOI 10 for 5 days. Infected MKN74 cell cultures had a significantly higher number of total mutations and transition mutations than non-infected control cell cultures. * denotes significantly different from untreated cells p<0.05.

We have previously shown that infection with pathogenic *H. pylori* strains induced mutations in the mitochondrial genome in human cell culture [36]. Given that infection with *E. faecalis* increase intracellular ROS we examined if it also caused genomic mtDNA instability. The overall frequency of mitochondrial D-loop region mutational events was significantly higher in MKN74 cell cultures infected with *E. faecalis* (45.5%) than in non-infected control cell cultures (23.0%; p<0.01) (Figure 3). Furthermore, infected cells showed a higher number of transitions (36.4%) than control cells (21.8%; p<0.05). Deletions (0% control/3.9% infected), insertions (1.1% control/2.6% infected), and transversions (0% control/2.6% infected), the latter typically seen as a consequence of ROS, were detected more frequently in infected cells. Thus infection with *E. faecalis* induces mutations in the gastric cells (Figure 3).

### Infection caused an NFκB dominated inflammatory response and a ROS response

**Figure 4:**
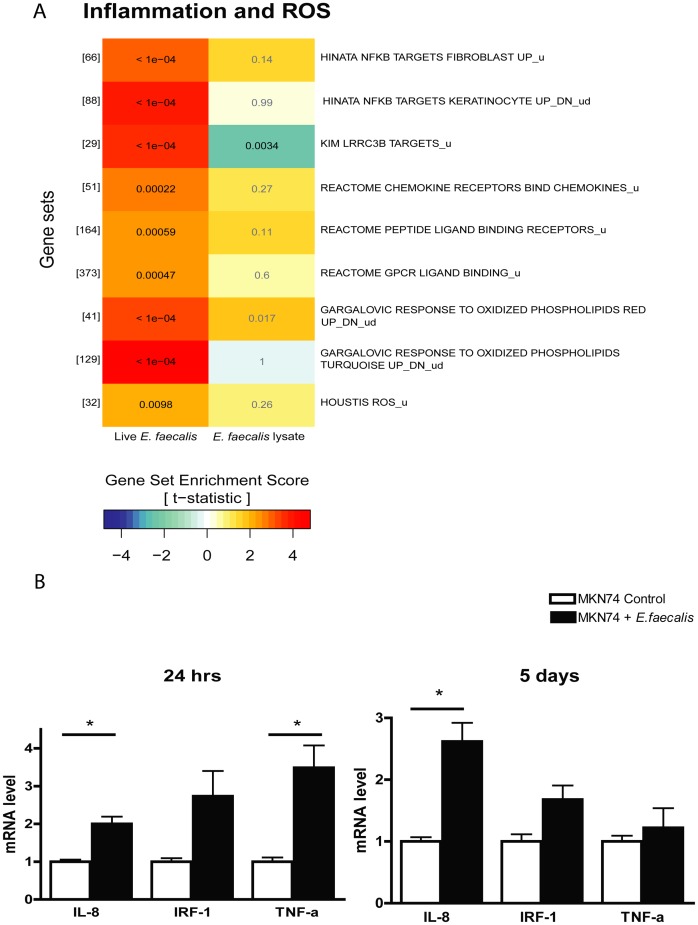
Infection stimulated an up-regulation of several transcripts involved in inflammatory and ROS response pathways. (A) A section of the GSA result. From the portion of gene sets significantly enriched in MKN74 cells infected for 24 hours, a subset of gene sets were manually selected by association with inflammatory response and response to ROS. The numbers in hard brackets indicate effective number of genes in the gene set. The title of each gene set corresponds to the title given on the MSigDB website. Numbers in colored boxes are adjusted p-values (q-values), black indicate statistical significance at 1% Fdr. (B) Characterization by qRT-PCR of transcripts coding for important cytokines and chemokines after *E. faecalis* infection for 24 hours (MOI50) and 5 days (MOI10). * denotes significantly different from untreated cells p<0.05.

**Figure 5:**
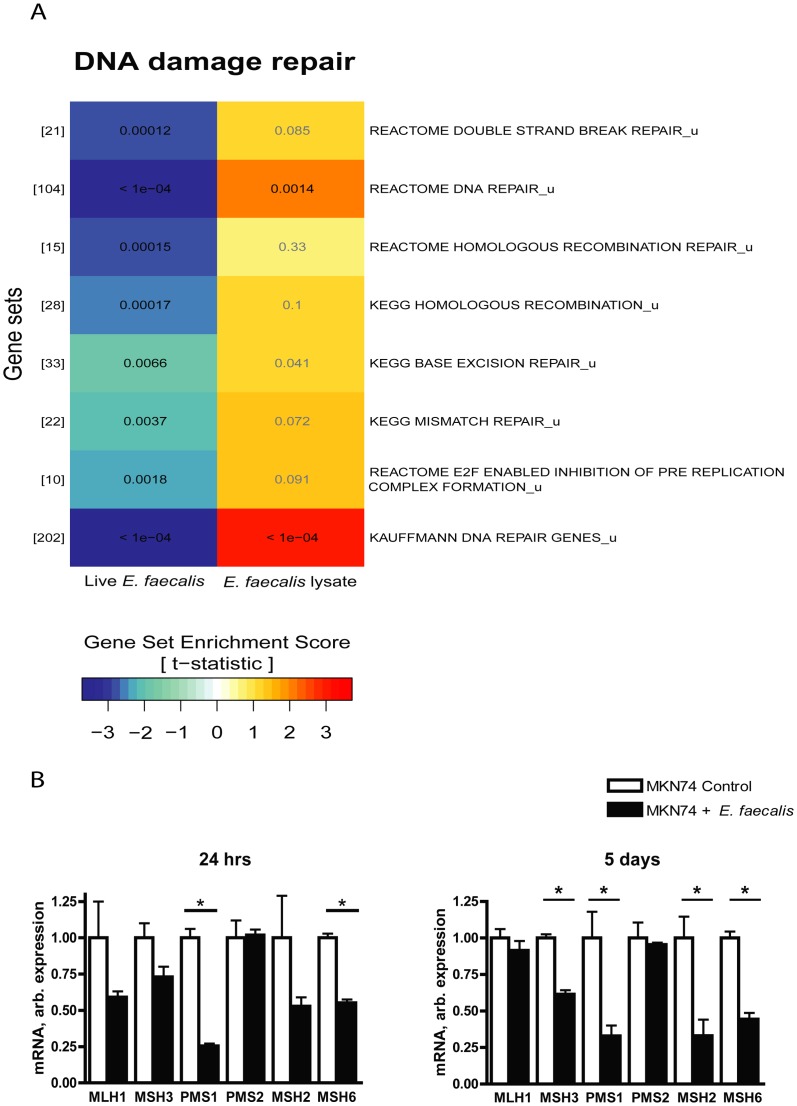
Infection stimulated an up-regulation of several transcripts involved in DNA damage response pathways. (A) A section of the GSA result after 24 hours of infection. Gene sets associated with DNA damage repair with same setup as figure 4 A. (B) Characterization of transcripts coding for important genes involved in MMR after *E. faecalis* infection for 24 hours (MOI50) and 5 days (MOI10). * denotes significantly different from untreated cells p<0.05.

**Figure 6:**
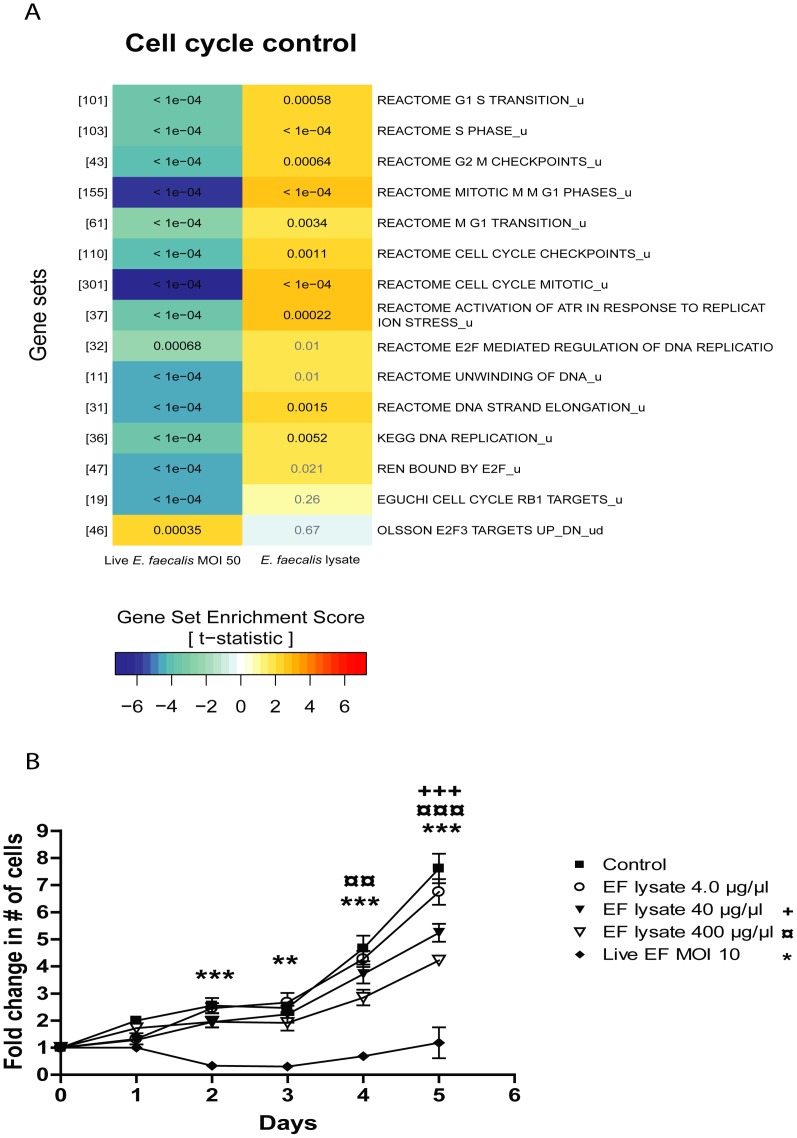
Infection caused a down-regulation of transcripts involved in cell cycle control pathways, and *E. faecalis* lysate slowed down cell proliferation. (A) A section of the GSA result after 24 hours of infection. Gene sets associated with cell cycle control with same setup as figure 4 A. (B) MKN74 growth assay. The fold change in number of cells relative to day zero is shown. The growth of MKN74 cells, infected with viable *E. faecalis* at a MOI of 10 or with different concentrations of bacterial lysate, was followed for a period of five days. Uninfected cells served as controls. Cell density was measured by crystal violet absorbance. ** and *** denotes significant difference p<0.01 and p<0.001 respectively.

To gain further biological insight in the cellular responses in gastric cells to *E. faecalis* infection we performed a microarray analysis examining the global changes in gene expression in cells infected with viable *E. faecalis* or treated with *E. faecalis* lysate. Rather than focusing on the expression of single genes we used GSA [30] to identify pathways and processes activated or inhibited by the infection. Focusing on sets of genes instead of individual genes increases robustness, sensitivity and biological relevance even for moderately regulated sets of genes. This is achieved by boosting the signal-to-noise ratio, making it possible to interpret modest changes in individual genes [37]. The GSA tested 2403 gene sets and resulted in 484 regulated pathways being statistically significant for either live infections and/or lysate stimuli (Figure S2). Focusing on those pathways statistically significant for the live infections, some of the gene sets judged to best characterize the current study were manually partitioned into three groups (Figures 4, 5 A and 6 A). Examining inflammation and ROS, *E. faecalis* infection caused a significant up-regulation of several pathways involved in immune responses, including clusters of chemokine receptors and chemokine signaling genes, genes involved in G-protein coupled receptor ligand binding, and NFκB activating genes (Figure 4, upper six gene sets). Additionally two gene sets comprising genes responding to pro-inflammatory oxidized phospholipids were strongly up-regulated. Oxidized phospholipids are produced by ROS and function in a pro-inflammatory manner [38], [39] (Figure 4, gene set seven and eight). Supporting the presence of ROS, a gene set of 30 genes known to be expressed in response to ROS was activated (Figure 4, bottom gene set). It is known that ROS are important for lipopolysaccharide (LPS)-driven production of several pro-inflammatory cytokines [40] however, gram-positive bacteria such as *E. faecalis* do not express LPS. We therefore examined how the expression of individual genes coding for pro-inflammatory cytokines and chemokines were effected in response to a non-LPS stimulated infection during 24 hour and 5 day infections (Table 1). Numerous genes involved in the NFκB inflammatory pathway were significantly up-regulated during *E. faecalis* infection including interleukin-1β (IL-1ß), interleukin-1 receptor-associated kinase 2 (IRAK2), VEGF, IL-8, IL-23α, IL-11, and Tumor Necrosis Factor-α (TNF-α) most of which can further propagate the NF-κB activation response [41]–[44]. Among these were several cytokines previously identified in *H. pylori* mediated infections, such as IL-8 and vascular endothelial growth factor (VEGF), both associated with angiogenesis and with advanced gastric cancer, IL-1ß which is a powerful inhibitor of acid secretion, and also TNF-α, a regulator of cell proliferation, differentiation and apoptosis [43]–[45]. In addition, the cytokine IL-23α which promotes tumor incidence and growth [46] and is highly produced in *H. pylori* colonized mucosa [47] was up-regulated along with, IL-11 which is a parietal cell cytokine that blocks gastric acid secretion, promotes atrophic gastritis and gastric tumorigenesis [48], [49]. Among other genes found to display high, and significant expressional levels during infection were IL-1α, IL-32, growth differentiation factor 15 (GDF15) and, chemokine (C-C motif) ligand 22 (CCL22). In addition, an up-regulation of superoxide dismutase 2 (SOD2), which converts superoxide to hydrogen peroxide, was seen. IL-8, Interferon regulatory factor 1 (IRF-1) and TNFα were validated by qRT-PCR (Figure 4 B).

**Table 1:** Genes involved in the inflammatory response of MKN74 cells identified by microarray analysis.

Gene	NCBI Reference ID	24 h fold change	Difference of means	P value 24 h	5 day fold change	Difference of means	P value 5 day
CCL22	NM_002990	2.7*	92	2.80E-05	6.0*	403	2.91E–07
GDF15	NM_004864	6.9*	5201	5.87E–08	−1.8*	5204	0.000363
IL1α	NM_000575	4.0*	1251	0.000829	−2.7*	1722	0.005858
IL1β	NM_000576	1.5*	45	0.001131	2.0*	154	2.29E–05
IL8	NM_000584	2.4*	2908	6.29E–05	2.9*	5203	1.44E–05
IL11	NM_000641	1.9*	266	0.002872	2.2*	160	0.000768
IL23α	NM_016584	3.8*	509	2.54E–06	4.0*	844	1.88E–06
IL32	NM_001012631	1.2	287	0.43468	3.7*	2180	8.73E–05
IRAK2	NM_001570	1.7*	265	2.62E–05	2.1*	511	1.99E–06
IRF1	NM_002198	1.1	10	0.45834	1.7*	55	0.005674
SOD2	NM_000636	1.9*	203	0.009899	2.2*	264	0.002617
TNFα	NM_006291	1.7*	58	0.00259	1.7*	134	0.003601
VEGFA	NM_001025366	3.9*	255	1.15E–08	−1.3*	130	0.00136

Selected genes are shown. The table shows expressional fold change of selected genes in MKN74 cells infected with *E. faecalis* for 24 hours and 5 days. Difference of means  =  difference in arbitrary expressional level of control cells and infected cells. *  =  P value <0.05.

### DNA damage repair are down regulated during *E. faecalis* infection

The pathway analysis indicated that the expression of genes involved in DNA damage repair was significantly down-regulated compared to uninfected cell cultures after 24 hours of infection (Figure 5A). qRT-PCR confirmed that the expression of several mismatch repair (MMR) genes were down-regulated after both 24 h and 5 days of *E. faecalis* infection (Figure 5, B). In 24 h infected cells PMS1 and MSH6 was significantly down-regulated (4 fold and 1.8 fold respectively; P<0.001). A significant down-regulation of MSH2 (3 fold), MSH3 (1.9 fold), MSH6 (2 fold), and PMS1 (3 fold) (p<0.05) (Figure 5, B) was observed in cells 5 days post infection. The expressional fold-changes of selected DNA damage repair genes after infection are shown in Table S3. These results suggest that *E. faecalis* infection down-regulates major DNA repair pathways resulting in genomic instability similar to infections with *H. pylori* [36], [50], [51].

### Infection by *E. faecalis* inhibits cell cycle control and growth of MKN74 cells

To evaluate the changes in cell growth induced by *E. faecalis* infection, we investigated the influence of the bacteria on the expression of cell cycle control genes and on MKN74 cell growth. Gene sets comprising cell cycle control and checkpoint genes were down-regulated after 24 hours of infection with viable bacteria suggesting a significant slowing down in cell proliferation but also a increased risk of mutations in new cells (Figure 6 A). In contrast, these pathways were up-regulated in cells infected with bacterial lysate suggesting that these cells were still dividing after 24 hours (Figure 6 A). We next measured cell proliferation for 5 days and found that viable bacteria caused MKN74 cell proliferation to slow down or stop completely (Figure 6 B). Cell proliferations were also affected when incubated with bacterial lysate. After 24 hours, lysate treated cells were still dividing (as also shown in Figure 6A), and until day 4 there was no distinction between uninfected cells and cells infected with lysate. However, on day 4 there were significantly less cells after incubation with bacterial lysate when compared to uninfected control cells. On day 5 a dose-response relationship between lysate concentration and cell growth was observed (Figure 6 B), showing that infections by *E. faecalis* interfere with cell growth. A similar result has been observed in human lymphocytes in which three days of incubation with *E. faecalis* lysate induced cell cycle arrest [52]. As this work was done in an isolated system, the reduced proliferation of infected cells could be attributed to the lack of growth factor stimulation by responding immune cells.

## Discussion

Microbial pathogens are estimated to cause approximately 1.2 million cases of cancer each year [5]. Human studies and several animal models have shown that achlorhydria allows for bacterial overgrowth of the stomach, which can cause chronic gastric inflammation, that develops into gastric cancer [20], [53], [54]. Supporting the etiologic role of other bacteria in gastric malignancies, are eradication studies of *H. pylori* infection which allow gastric colonization by intestinal bacteria [55], and a subsequent gastric cancer development [56]. This suggests that other players than *H. pylori* may be involved in inflammation driven gastric malignancies. The molecular events by which chronic gastric infection with these bacteria affect cancer cells and cause damage to these cells has been poorly understood.

We found that infection with *E. faecalis* induced an intracellular production of ROS. The oxidative phosphorylation by the mitochondria was low after infection, showing that ROS production was mainly generated in an oxphos-independent way within the infected cells. Intracellular oxphos-independent ROS can be produced in epithelial cells by non-phagocytic NADPH (NOX) oxidases in response to e.g. cytokine stimulation [57], and are the major non-mitochondrial sources of ROS [58]. These NOX enzymes transport electrons across plasma membranes, generating superoxide and other ROS [59]. In human epithelial cells two NOX homologus (NOX2 and NOX5) are found [60]. In our study these enzymes were not transcriptionally up-regulated, however it cannot be ruled out that the elevated ROS levels was a consequence of an activation of these enzymes, although this needs further investigation. ROS affect several biological processes such as cellular signaling and aging, and plays an important role in host defense against invading microorganisms, however, an overproduction of ROS is cytotoxic and carcinogenic [61]. One of the mechanisms behind *H. pylori*-induced gastric injury is production of ROS by infiltrating neutrophils in the infected tissues [62]. However, *H. pylori* can also stimulate ROS production in gastric epithelial cells in the absence of inflammatory cells [63]. We have now shown that *E. faecalis* also induce gastric epithelial cells to produce ROS. Similar observations in colonic epithelial cells (for review see [64]) suggest that *E. faecalis* associated gastric pathogenicity in part operates via a more common mechanism for epithelial cell damage.

We also found induction of mutations in the mitochondrial D-loop region in cells infected with *E. faecalis*. Mutations in the mitochondrial genome are common in a broad range of cancers, including gastric cancer and may have severe effects on the mitochondrial oxidative phosphorylation and consequently cellular metabolism [65], [66]. The D-loop mutations in the *E. faecalis* infected cells of this study were mostly transitions. Transition mutations have previously been shown to be the major mutational events in mtDNA of gastric carcinomas from human tumors, as well as in *H. pylori* infected cells [36], [65].

Besides initiating intracellular ROS production and mtDNA instability, we have demonstrated that *E. faecalis* infection also lead to reduced expression of several genes involved in DNA repair, e.g. MMR gene expression. We have previously shown a similar effect in the expression of MMR genes in response to *H. pylori* infection [36]. *In vivo* studies on *H. pylori* infection has furthermore shown impaired MMR [50] and increased genetic instability [36] that predisposes to gastric cancer. Thus by disrupting this mechanism *E. faecalis* contributes to damage of adenocarcinoma cells. In our experiments we further see a decrease in the expression of genes involved in important cell cycle checkpoint pathways creating an increased risk of the introduction of new mutations.

The mucus cells lining the intestinal tract have some capabilities of an immune system, e.g. the ability to sense and respond to foreign pathogens and to express and secrete cytokines [45]. We found that infected MKN74 cells with *E. faecalis* had an up-regulated expression of several NFκB regulated pro-inflammatory cytokines and chemokines. NF-κB is one of the key transcription factors in cancer-related inflammation [67]. During gastric infection there is a robust recruitment of lymphocytes and neutrophils from which many cytokines are secreted. However, not all cytokines are secreted by the immune cells. Mueller et al showed that mucus cells alone can secrete pro-inflammatory cytokines and mucosal defense responses [45] when infected with *H. Pylori*. We found several similarities between infections by *E. faecalis* and *H. pylori* in the cytokine and chemokine responses of the infected gastric cells including activation of cytokines known to stimulate NF-κB activation.

**Figure 7:**
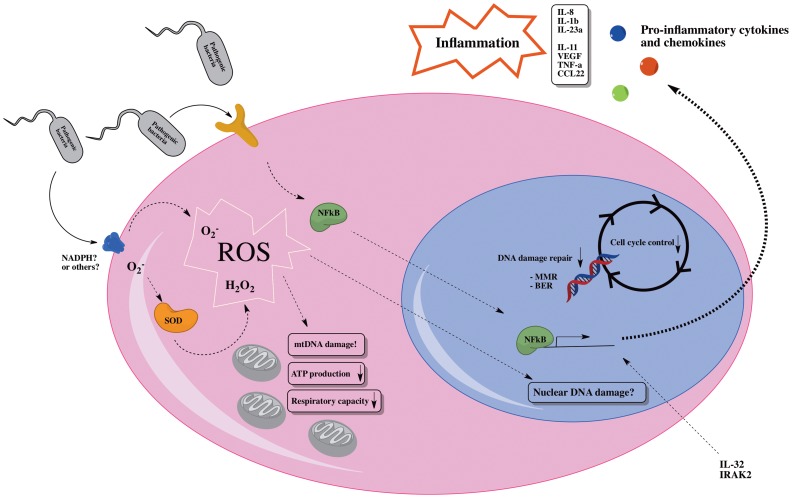
Proposed model for how chronic infection by pathogenic bacteria affects gastric epithelial cells.

In summary, the development of gastric malignancy is strongly associated with microbial infections especially by *H. pylori*, which chronically infects the gastric mucosa of more than half the world's population. We show that infection by *E. faecalis* induces an intracellular ROS production independent of oxidative phosphorylation whilst damaging the mitochondrial genome in gastric cells. The bacteria also induce an NF-κB driven pro-inflammatory response and impair DNA damage response and cell cycle control gene expression. We find many similarities between infection by *E. faecalis* and *H. pylori* and we therefore propose that infection with pathogenic bacteria other than *H. pylori* can induce cancer promoting events resulting in further damage to adenocarcinoma cells in the stomach (Figure 7). Some of the mechanisms by which chronic bacterial infection induce gastric cancer are surely pathogen specific such as the cytotoxin-associated gene (cag) and vacuolating cytotoxin gene (vacA) from *H. pylori* (for review see [68]) where as others seem to be linked to the general way epithelial cells responds to chronic infections. We propose a common link in bacterial induced pathogenicity by which the infection stimulates a general activation of pro-inflammatory cytokines and ROS by the cells themselves, making the epithelial cells harmful to themselves. Furthermore, the bacterial infection gives DNA damage and impairs the cells ability to repair this damage. Knowledge of pathogen specific mechanisms underlying gastric infection and malignancy are important as this can help development of pathogen specific therapies. Also, identification of common mechanisms by which bacteria damage cells is important as this can point to targets shared by several pathogens.

## Supporting Information

Figure S1 **Conversion of OD_550_ measurements to CFU/ml.** (A) Bacteria were grown in RPMI 1640 medium and OD_550_ measurements were taken at various time points. (B) and (C) Dilutions of 10^−3^–10^−7^ were made for every OD_550_ measurement in the exponential growth phase, and 100 µl of each dilution was plated in duplicates on blood agar plates. Plates with approximately 50–200 CFU were selected and the number of CFU per plate was counted. An average cell count was taken, and correlated to an OD_550_ measurement of 1. Finally an average of the correlated values was taken, and used for further calculations in the infection.
(PDF)

Figure S2 **Total GSA list of 24 hour infections (Live** ***E. faecalis*** **and** ***E. faecalis*** **lysate).**
(PDF)

Table S1 **Primers.**
(DOCX)

Table S2 **Expressional levels and fold change of the seven NADPH family members in MKN74 cell cultures during 24 hrs and 5 days of infection with** ***E. faecalis*** **.** The phagocyte NADPH oxidase (NOX2) has six homologs: NOX1, NOX3-5 and DUOX1-2, constituting the NOX family of NADPH oxidases.
(DOCX)

Table S3 **DNA damage repair genes identified by microarray analysis.** Selected genes are shown. The table shows expressional fold change of these genes in MKN74 cells infected with *E. faecalis* for 24 hours and 5 days. Difference of means  =  difference in arbitrary expressional level of control cells and infected cells. *  =  P value <0.05.
(DOCX)

File S1 (DOCX)

File S2 (DOCX)
