# Supplementary material for: Enterococcus faecalis Infection Causes Inflammation, Intracellular Oxphos-Independent ROS Production, and DNA Damage in Human Gastric Cancer Cells
Source: PLoS One. 2013 Apr 30;8(4):e63147. doi: 10.1371/journal.pone.0063147 (PMC3639970; doi:10.1371/journal.pone.0063147)
Supplement: Figure S1 — Conversion of OD550 measurements to CFU/ml. (A) Bacteria were grown in RPMI 1640 medium and OD550 measurements were taken at various time points. (B) and (C) Dilutions of 10−3–10−7 were made for every OD550 measurement in the exponential growth phase, and 100 µl of each dilution was plated in duplicates on blood agar plates. Plates with approximately 50–200 CFU were selected and the number of CFU per plate was counted. An average cell count was taken, and correlated to an OD550 measurement of 1. Finally an average of the correlated values was taken, and used for further calculations in the infection. (PDF) [file pone.0063147.s001.pdf]

Figure S1

A

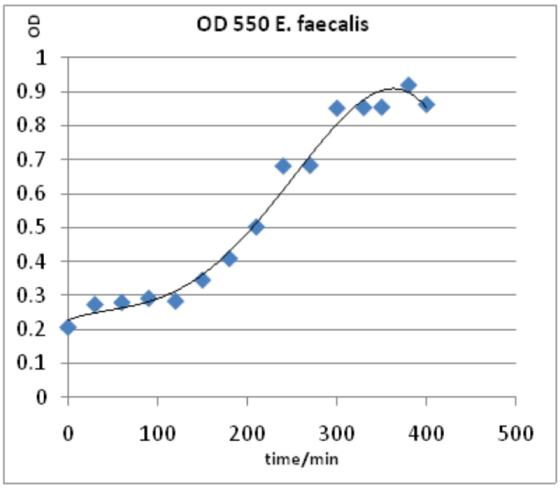

B

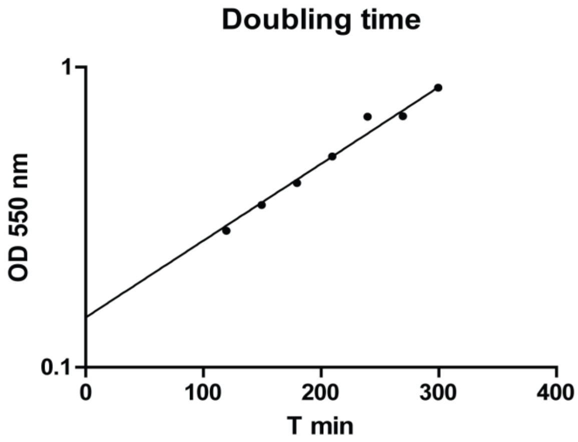

C

OD_{550}	Dilution	CFU count plate #1	CFU count plate # 2	Average # CFU	*1 OD_{550}= CFU/ml
0,124	10^{-5}	41	45	43	$0,35\cdot10^{9}$ CFU/ml
0,207	10^{-5}	92	85	89	$0,43\cdot10^{9}$ CFU/ml
0,346	10^{-5}	182	233	208	$0,60\cdot10^{9}$ CFU/ml
0,513	10^{-6}	29	39	34	$0,66\cdot10^{9}$ CFU/ml
0,681	10^{-6}	90	71	81	$1,18\cdot10^{9}$ CFU/ml
				Average: 1 OD_{550} = $0,64\cdot10^{9}$ CFU/ml	

*Calculations of CFU/ml to an OD_{550} measurement of 1 were done as follows:

1) Correlating the OD_{550} measurements to actual number of CFU/ml:

$$\frac{\text{Average \# CFU} \cdot \text{Dilution factor}}{\text{Plated volume (ml)}} = \text{Actual \# CFU/ml}$$

2) Correlating the actual number of CFU/ml with an OD_{550} measurement of 1:

$$\frac{\text{Actual \# CFU/ml}}{\text{Measured value of } OD_{550}} = 1\ OD_{550}$$
