# Supplementary material for: Enterococcus faecalis Infection Causes Inflammation, Intracellular Oxphos-Independent ROS Production, and DNA Damage in Human Gastric Cancer Cells
Source: PLoS One. 2013 Apr 30;8(4):e63147. doi: 10.1371/journal.pone.0063147 (PMC3639970; doi:10.1371/journal.pone.0063147)
Supplement: Figure S2 — Total GSA list of 24 hour infections (Live E. faecalis and E. faecalis lysate). (PDF) [file pone.0063147.s002.pdf]

Figure S2

Significant gene sets (1% Fdr) (part 1 of 9)
[q−values]

Gene set 1−−60 of 484

	Enterococcus live.24h.MOI 50	Enterococcus lysate.24h.lysate	
[771]	< 1e−04	0.19	TIEN_INTESTINE_PROBIOTICS_24HR_UP_DN_ud
[140]	< 1e−04	0.00019	ROSTY_CERVICAL_CANCER_PROLIFERATION_CLUSTER_u
[428]	< 1e−04	< 1e−04	PUJANA_BRCA2_PCC_NETWORK_u
[444]	< 1e−04	< 1e−04	TOYOTA_TARGETS_OF_MIR34B_AND_MIR34C_u
[773]	< 1e−04	< 1e−04	PUJANA_CHEK2_PCC_NETWORK_u
[301]	< 1e−04	< 1e−04	REACTOME_CELL_CYCLE_MITOTIC_u
[781]	< 1e−04	< 1e−04	WEI_MYCN_TARGETS_WITH_E_BOX_u
[642]	< 1e−04	0.0031	BENPORATH_CYCLING_GENES_u
[661]	< 1e−04	< 1e−04	BASAKI_YBX1_TARGETS_UP_DN_ud
[207]	< 1e−04	< 1e−04	SOTIRIOU_BREAST_CANCER_GRADE_1_VS_3_UP_DN_ud
[278]	< 1e−04	0.009	GRAHAM_CML_DIVIDING_VS_NORMAL_QUIESCENT_UP_DN_ud
[155]	< 1e−04	< 1e−04	REACTOME_MITOTIC_M_M_G1_PHASES_u
[892]	< 1e−04	< 1e−04	BERENJENO_TRANSFORMED_BY_RHOA_UP_DN_ud
[167]	< 1e−04	< 1e−04	PUJANA_XPRSS_INT_NETWORK_u
[817]	< 1e−04	< 1e−04	CAIRO_HEPATOBLASTOMA_CLASSES_UP_DN_ud
[30]	< 1e−04	8e−04	REACTOME_ACTIVATION_OF_THE_PRE_REPLICATIVE_COMPLEX_u
[134]	< 1e−04	0.06	HOFFMANN_LARGE_TO_SMALL_PRE_BII_LYMPHOCYTE_UP_DN_u d
[49]	< 1e−04	2e−04	SONG_TARGETS_OF_IE86_CMV_PROTEIN_u
[455]	< 1e−04	< 1e−04	SHEDDEN_LUNG_CANCER_POOR_SURVIVAL_A6_u
[92]	< 1e−04	0.12	WHITEFORD_PEDIATRIC_CANCER_MARKERS_u
[285]	< 1e−04	< 1e−04	FOURNIER_ACINAR_DEVELOPMENT_LATE_2_u
[90]	< 1e−04	0.00027	REACTOME_MITOTIC_PROMETAPHASE_u
[789]	< 1e−04	< 1e−04	VECCHI_GASTRIC_CANCER_EARLY_UP_DN_ud
[73]	< 1e−04	0.19	KANG_DOXORUBICIN_RESISTANCE_UP_DN_ud
[846]	< 1e−04	< 1e−04	GEORGES_TARGETS_OF_MIR192_AND_MIR215_u
[47]	< 1e−04	0.021	REN_BOUND_BY_E2F_u
[79]	< 1e−04	< 1e−04	REACTOME_OLFACTORY_SIGNALING_PATHWAY_b
[331]	< 1e−04	< 1e−04	WONG_EMBRYONIC_STEM_CELL_CORE_u
[237]	< 1e−04	< 1e−04	FERREIRA_EWINGS_SARCOMA_UNSTABLE_VS_STABLE_UP_DN_u d
[537]	< 1e−04	< 1e−04	ZHANG_BREAST_CANCER_PROGENITORS_UP_DN_ud
[93]	< 1e−04	< 1e−04	PUJANA_BRCA_CENTERED_NETWORK_u
[375]	< 1e−04	< 1e−04	BENPORATH_ES_1_u
[912]	< 1e−04	< 1e−04	NAKAMURA_TUMOR_ZONE_PERIPHERAL_VS_CENTRAL_UP_DN_ud
[31]	< 1e−04	0.0015	REACTOME_DNA_STRAND_ELONGATION_u
[11]	< 1e−04	0.01	REACTOME_UNWINDING_OF_DNA_u
[292]	< 1e−04	< 1e−04	MUELLER_PLURINET_u
[19]	< 1e−04	0.26	EGUCHI_CELL_CYCLE_RB1_TARGETS_u
[127]	< 1e−04	0.5	LEE_EARLY_T_LYMPHOCYTE_UP_DN_ud
[156]	< 1e−04	< 1e−04	ELVIDGE_HIF1A_TARGETS_UP_DN_ud
[76]	< 1e−04	0.00027	REACTOME_DNA_REPLICATION_PRE_INITIATION_u
[537]	< 1e−04	0.051	JAATINEN_HEMATOPOIETIC_STEM_CELL_UP_DN_ud
[124]	< 1e−04	< 1e−04	DAZARD_UV_RESPONSE_CLUSTER_G6_u
[35]	< 1e−04	1	AMUNDSON_GAMMA_RADIATION_RESPONSE_u
[841]	< 1e−04	< 1e−04	WEST_ADRENOCORTICAL_TUMOR_UP_DN_ud
[50]	< 1e−04	0.047	CHANG_CYCLING_GENES_u
[43]	< 1e−04	0.00064	REACTOME_G2_M_CHECKPOINTS_u
[205]	< 1e−04	< 1e−04	WINNEPENNINCKX_MELANOMA_METASTASIS_DN_UP_du
[104]	< 1e−04	0.00021	PYEON_HPV_POSITIVE_TUMORS_UP_DN_ud
[110]	< 1e−04	0.0011	REACTOME_CELL_CYCLE_CHECKPOINTS_u
[138]	< 1e−04	0.00043	REACTOME_PROCESSING_OF_CAPPED_INTRON_CONTAINING_PR E_MRNA_u
[147]	< 1e−04	< 1e−04	BENPORATH_PROLIFERATION_u
[126]	< 1e−04	< 1e−04	KAUFFMANN_DNA_REPLICATION_GENES_u
[102]	< 1e−04	0.25	MORI_LARGE_PRE_BII_LYMPHOCYTE_UP_DN_ud
[64]	< 1e−04	0.0089	PUJANA_BREAST_CANCER_WITH_BRCA1_MUTATED_UP_DN_ud
[37]	< 1e−04	0.00022	REACTOME_ACTIVATION_OF_ATR_IN_RESPONSE_TO_REPLICAT ION_STRESS_u
[33]	< 1e−04	0.0059	FARMER_BREAST_CANCER_CLUSTER_2_u
[36]	< 1e−04	0.0052	KEGG_DNA_REPLICATION_u
[101]	< 1e−04	0.00058	REACTOME_G1_S_TRANSITION_u
[202]	< 1e−04	< 1e−04	KAUFFMANN_DNA_REPAIR_GENES_u
[94]	< 1e−04	0.001	REACTOME_METABOLISM_OF_RNA_u

Gene Set Enrichment Score
[t−statistic]

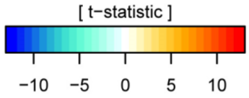

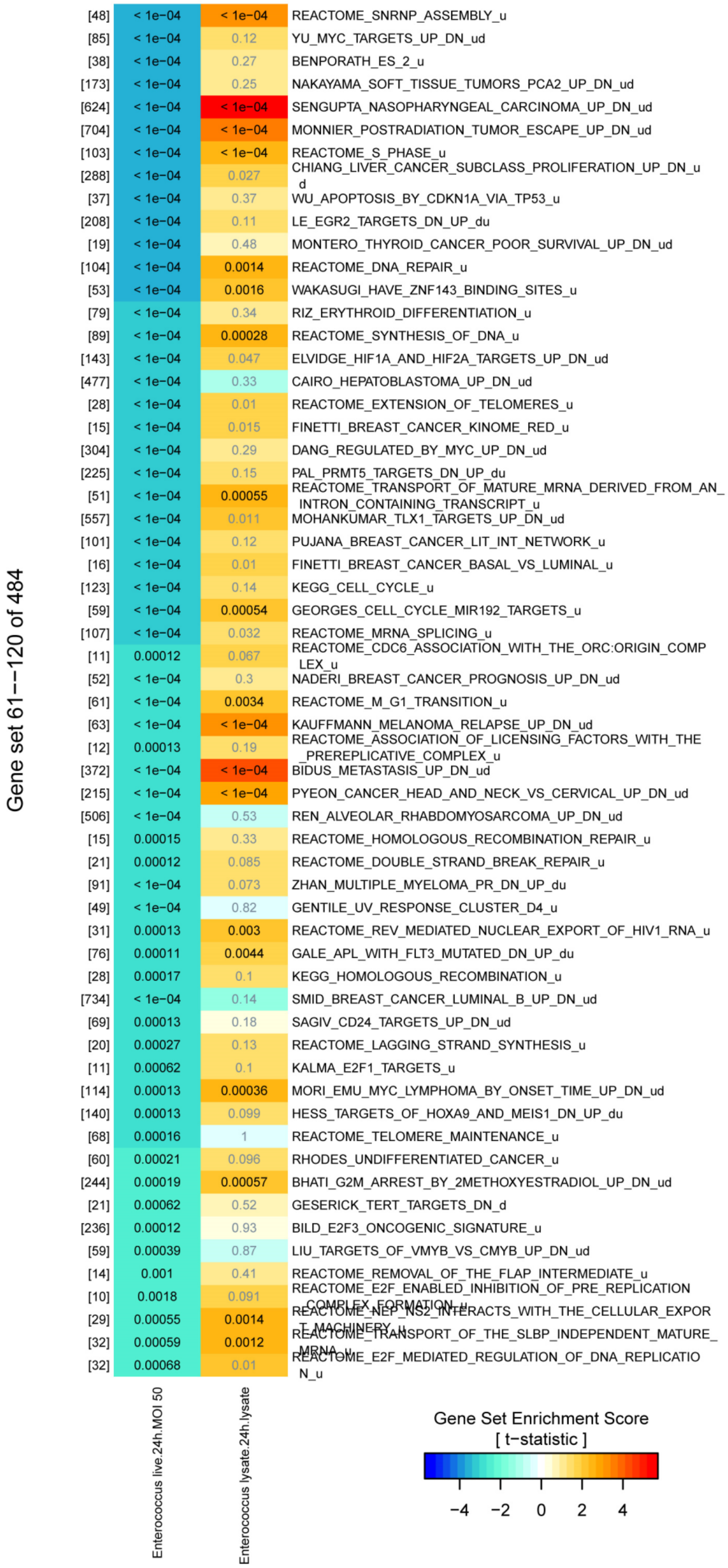
Gene set 61−−120 of 484
[48] < 1e−04 < 1e−04 REACTOME_SNRNP_ASSEMBLY_u
[85] < 1e−04 0.12 YU_MYC_TARGETS_UP_DN_ud
[38] < 1e−04 0.27 BENPORATH_ES_2_u
[173] < 1e−04 0.25 NAKAYAMA_SOFT_TISSUE_TUMORS_PCA2_UP_DN_ud
[624] < 1e−04 < 1e−04 SENGUPTA_NASOPHARYNGEAL_CARCINOMA_UP_DN_ud
[704] < 1e−04 < 1e−04 MONNIER_POSTRADIATION_TUMOR_ESCAPE_UP_DN_ud
[103] < 1e−04 < 1e−04 REACTOME_S_PHASE_u
[288] < 1e−04 0.027 CHIANG_LIVER_CANCER_SUBCLASS_PROLIFERATION_UP_DN_ud
[37] < 1e−04 0.37 WU_APOPTOSIS_BY_CDKN1A_VIA_TP53_u
[208] < 1e−04 0.11 LE_EGR2_TARGETS_DN_UP_du
[19] < 1e−04 0.48 MONTERO_THYROID_CANCER_POOR_SURVIVAL_UP_DN_ud
[104] < 1e−04 0.0014 REACTOME_DNA_REPAIR_u
[53] < 1e−04 0.0016 WAKASUGI_HAVE_ZNF143_BINDING_SITES_u
[79] < 1e−04 0.34 RIZ_ERYTHROID_DIFFERENTIATION_u
[89] < 1e−04 0.00028 REACTOME_SYNTHESIS_OF_DNA_u
[143] < 1e−04 0.047 ELVIDGE_HIF1A_AND_HIF2A_TARGETS_UP_DN_ud
[477] < 1e−04 0.33 CAIRO_HEPATOBLASTOMA_UP_DN_ud
[28] < 1e−04 0.01 REACTOME_EXTENSION_OF_TELOMERES_u
[15] < 1e−04 0.015 FINETTI_BREAST_CANCER_KINOME_RED_u
[304] < 1e−04 0.29 DANG_REGULATED_BY_MYC_UP_DN_ud
[225] < 1e−04 0.15 PAL_PRMT5_TARGETS_DN_UP_du
[51] < 1e−04 0.00055 REACTOME_TRANSPORT_OF_MATURE_MRNA_DERIVED_FROM_AN_INTRON_CONTAINING_TRANSCRIPT_u
[557] < 1e−04 0.011 MOHANKUMAR_TLX1_TARGETS_UP_DN_ud
[101] < 1e−04 0.12 PUJANA_BREAST_CANCER_LIT_INT_NETWORK_u
[16] < 1e−04 0.01 FINETTI_BREAST_CANCER_BASAL_VS_LUMINAL_u
[123] < 1e−04 0.14 KEGG_CELL_CYCLE_u
[59] < 1e−04 0.00054 GEORGES_CELL_CYCLE_MIR192_TARGETS_u
[107] < 1e−04 0.032 REACTOME_MRNA_SPLICING_u
[11] 0.00012 0.067 REACTOME_CDC6_ASSOCIATION_WITH_THE_ORC:ORIGIN_COMPLEX_u
[52] < 1e−04 0.3 NADERI_BREAST_CANCER_PROGNOSIS_UP_DN_ud
[61] < 1e−04 0.0034 REACTOME_M_G1_TRANSITION_u
[63] < 1e−04 < 1e−04 KAUFFMANN_MELANOMA_RELAPSE_UP_DN_ud
[12] 0.00013 0.19 REACTOME_ASSOCIATION_OF_LICENSING_FACTORS_WITH_THE_PREREPLICATIVE_COMPLEX_u
[372] < 1e−04 < 1e−04 BIDUS_METASTASIS_UP_DN_ud
[215] < 1e−04 < 1e−04 PYEON_CANCER_HEAD_AND_NECK_VS_CERVICAL_UP_DN_ud
[506] < 1e−04 0.53 REN_ALVEOLAR_RHABDOMYOSARCOMA_UP_DN_ud
[15] 0.00015 0.33 REACTOME_HOMOLOGOUS_RECOMBINATION_REPAIR_u
[21] 0.00012 0.085 REACTOME_DOUBLE_STRAND_BREAK_REPAIR_u
[91] < 1e−04 0.073 ZHAN_MULTIPLE_MYELOMA_PR_DN_UP_du
[49] < 1e−04 0.82 GENTILE_UV_RESPONSE_CLUSTER_D4_u
[31] 0.00013 0.003 REACTOME_REV_MEDIATED_NUCLEAR_EXPORT_OF_HIV1_RNA_u
[76] 0.00011 0.0044 GALE_APL_WITH_FLT3_MUTATED_DN_UP_du
[28] 0.00017 0.1 KEGG_HOMOLOGOUS_RECOMBINATION_u
[734] < 1e−04 0.14 SMID_BREAST_CANCER_LUMINAL_B_UP_DN_ud
[69] 0.00013 0.18 SAGIV_CD24_TARGETS_UP_DN_ud
[20] 0.00027 0.13 REACTOME_LAGGING_STRAND_SYNTHESIS_u
[11] 0.00062 0.1 KALMA_E2F1_TARGETS_u
[114] 0.00013 0.00036 MORI_EMU_MYC_LYMPHOMA_BY_ONSET_TIME_UP_DN_ud
[140] 0.00013 0.099 HESS_TARGETS_OF_HOXA9_AND_MEIS1_DN_UP_du
[68] 0.00016 1 REACTOME_TELOMERE_MAINTENANCE_u
[60] 0.00021 0.096 RHODES_UNDIFFERENTIATED_CANCER_u
[244] 0.00019 0.00057 BHATI_G2M_ARREST_BY_2METHOXYESTRADIOL_UP_DN_ud
[21] 0.00062 0.52 GESERICK_TERT_TARGETS_DN_d
[236] 0.00012 0.93 BILD_E2F3_ONCOGENIC_SIGNATURE_u
[59] 0.00039 0.87 LIU_TARGETS_OF_VMYB_VS_CMYB_UP_DN_ud
[14] 0.001 0.41 REACTOME_REMOVAL_OF_THE_FLAP_INTERMEDIATE_u
[10] 0.0018 0.091 REACTOME_E2F_ENABLED_INHIBITION_OF_PRE_REPLICATION_COMPLEX_FORMATION_u
[29] 0.00055 0.0014 REACTOME_NEP_NS2_INTERACTS_WITH_THE_CELLULAR_EXPORT_MACHINERY_u
[32] 0.00059 0.0012 REACTOME_TRANSPORT_OF_THE_SLBP_INDEPENDENT_MATURE_MRNA_u
[32] 0.00068 0.01 REACTOME_E2F_MEDIATED_REGULATION_OF_DNA_REPLICATION_u
Enterococcus live.24h.MOI 50
Enterococcus lysate.24h.lysate
Gene Set Enrichment Score
[t−statistic]
−4 −2 0 2 4

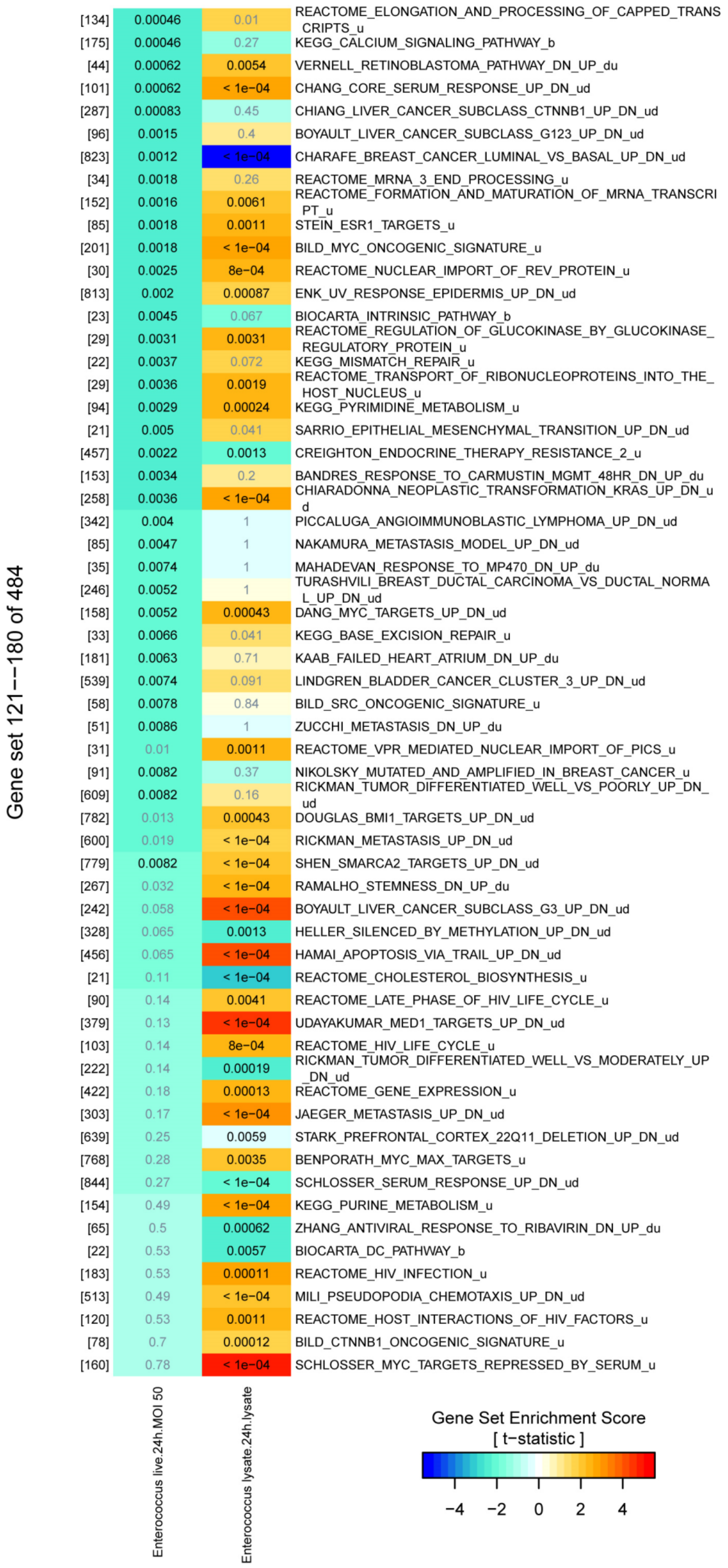
Gene set 121 -- 180 of 484
[134] 0.00046 0.01 REACTOME_ELONGATION_AND_PROCESSING_OF_CAPPED_TRANSCRIPTS_u
[175] 0.00046 0.27 KEGG_CALCIUM_SIGNALING_PATHWAY_b
[44] 0.00062 0.0054 VERNELL_RETINOBLASTOMA_PATHWAY_DN_UP_du
[101] 0.00062 < 1e−04 CHANG_CORE_SERUM_RESPONSE_UP_DN_ud
[287] 0.00083 0.45 CHIANG_LIVER_CANCER_SUBCLASS_CTNNB1_UP_DN_ud
[96] 0.0015 0.4 BOYAULT_LIVER_CANCER_SUBCLASS_G123_UP_DN_ud
[823] 0.0012 < 1e−04 CHARAFE_BREAST_CANCER_LUMINAL_VS_BASAL_UP_DN_ud
[34] 0.0018 0.26 REACTOME_MRNA_3_END_PROCESSING_u
[152] 0.0016 0.0061 REACTOME_FORMATION_AND_MATURATION_OF_MRNA_TRANSCRIPT_u
[85] 0.0018 0.0011 STEIN_ESR1_TARGETS_u
[201] 0.0018 < 1e−04 BILD_MYC_ONCOGENIC_SIGNATURE_u
[30] 0.0025 8e−04 REACTOME_NUCLEAR_IMPORT_OF_REV_PROTEIN_u
[813] 0.002 0.00087 ENK_UV_RESPONSE_EPIDERMIS_UP_DN_ud
[23] 0.0045 0.067 BIOCARTA_INTRINSIC_PATHWAY_b
[29] 0.0031 0.0031 REACTOME_REGULATION_OF_GLUCOKINASE_BY_GLUCOKINASE_REGULATORY_PROTEIN_u
[22] 0.0037 0.072 KEGG_MISMATCH_REPAIR_u
[29] 0.0036 0.0019 REACTOME_TRANSPORT_OF_RIBONUCLEOPROTEINS_INTO_THE_HOST_NUCLEUS_u
[94] 0.0029 0.00024 KEGG_PYRIMIDINE_METABOLISM_u
[21] 0.005 0.041 SARRIO_EPITHELIAL_MESENCHYMAL_TRANSITION_UP_DN_ud
[457] 0.0022 0.0013 CREIGHTON_ENDOCRINE_THERAPY_RESISTANCE_2_u
[153] 0.0034 0.2 BANDRES_RESPONSE_TO_CARMUSTIN_MGMT_48HR_DN_UP_du
[258] 0.0036 < 1e−04 CHIARADONNA_NEOPLASTIC_TRANSFORMATION_KRAS_UP_DN_ud
[342] 0.004 1 PICCALUGA_ANGIOIMMUNOBLASTIC_LYMPHOMA_UP_DN_ud
[85] 0.0047 1 NAKAMURA_METASTASIS_MODEL_UP_DN_ud
[35] 0.0074 1 MAHADEVAN_RESPONSE_TO_MP470_DN_UP_du
[246] 0.0052 1 TURASHVILI_BREAST_DUCTAL_CARCINOMA_VS_DUCTAL_NORMAL_UP_DN_ud
[158] 0.0052 0.00043 DANG_MYC_TARGETS_UP_DN_ud
[33] 0.0066 0.041 KEGG_BASE_EXCISION_REPAIR_u
[181] 0.0063 0.71 KAAB_FAILED_HEART_ATRIUM_DN_UP_du
[539] 0.0074 0.091 LINDGREN_BLADDER_CANCER_CLUSTER_3_UP_DN_ud
[58] 0.0078 0.84 BILD_SRC_ONCOGENIC_SIGNATURE_u
[51] 0.0086 1 ZUCCHI_METASTASIS_DN_UP_du
[31] 0.01 0.0011 REACTOME_VPR_MEDIATED_NUCLEAR_IMPORT_OF_PICS_u
[91] 0.0082 0.37 NIKOLSKY_MUTATED_AND_AMPLIFIED_IN_BREAST_CANCER_u
[609] 0.0082 0.16 RICKMAN_TUMOR_DIFFERENTIATED_WELL_VS_POORLY_UP_DN_ud
[782] 0.013 0.00043 DOUGLAS_BMI1_TARGETS_UP_DN_ud
[600] 0.019 < 1e−04 RICKMAN_METASTASIS_UP_DN_ud
[779] 0.0082 < 1e−04 SHEN_SMARCA2_TARGETS_UP_DN_ud
[267] 0.032 < 1e−04 RAMALHO_STEMNESS_DN_UP_du
[242] 0.058 < 1e−04 BOYAULT_LIVER_CANCER_SUBCLASS_G3_UP_DN_ud
[328] 0.065 0.0013 HELLER_SILENCED_BY_METHYLATION_UP_DN_ud
[456] 0.065 < 1e−04 HAMAI_APOPTOSIS_VIA_TRAIL_UP_DN_ud
[21] 0.11 < 1e−04 REACTOME_CHOLESTEROL_BIOSYNTHESIS_u
[90] 0.14 0.0041 REACTOME_LATE_PHASE_OF_HIV_LIFE_CYCLE_u
[379] 0.13 < 1e−04 UDAYAKUMAR_MED1_TARGETS_UP_DN_ud
[103] 0.14 8e−04 REACTOME_HIV_LIFE_CYCLE_u
[222] 0.14 0.00019 RICKMAN_TUMOR_DIFFERENTIATED_WELL_VS_MODERATELY_UP_DN_ud
[422] 0.18 0.00013 REACTOME_GENE_EXPRESSION_u
[303] 0.17 < 1e−04 JAEGER_METASTASIS_UP_DN_ud
[639] 0.25 0.0059 STARK_PREFRONTAL_CORTEX_22Q11_DELETION_UP_DN_ud
[768] 0.28 0.0035 BENPORATH_MYC_MAX_TARGETS_u
[844] 0.27 < 1e−04 SCHLOSSER_SERUM_RESPONSE_UP_DN_ud
[154] 0.49 < 1e−04 KEGG_PURINE_METABOLISM_u
[65] 0.5 0.00062 ZHANG_ANTIVIRAL_RESPONSE_TO_RIBAVIRIN_DN_UP_du
[22] 0.53 0.0057 BIOCARTA_DC_PATHWAY_b
[183] 0.53 0.00011 REACTOME_HIV_INFECTION_u
[513] 0.49 < 1e−04 MILI_PSEUDOPODIA_CHEMOTAXIS_UP_DN_ud
[120] 0.53 0.0011 REACTOME_HOST_INTERACTIONS_OF_HIV_FACTORS_u
[78] 0.7 0.00012 BILD_CTNNB1_ONCOGENIC_SIGNATURE_u
[160] 0.78 < 1e−04 SCHLOSSER_MYC_TARGETS_REPRESSED_BY_SERUM_u
Enterococcus live.24h.MOI 50
Enterococcus lysate.24h.lysate
Gene Set Enrichment Score
[t−statistic]
−4 −2 0 2 4

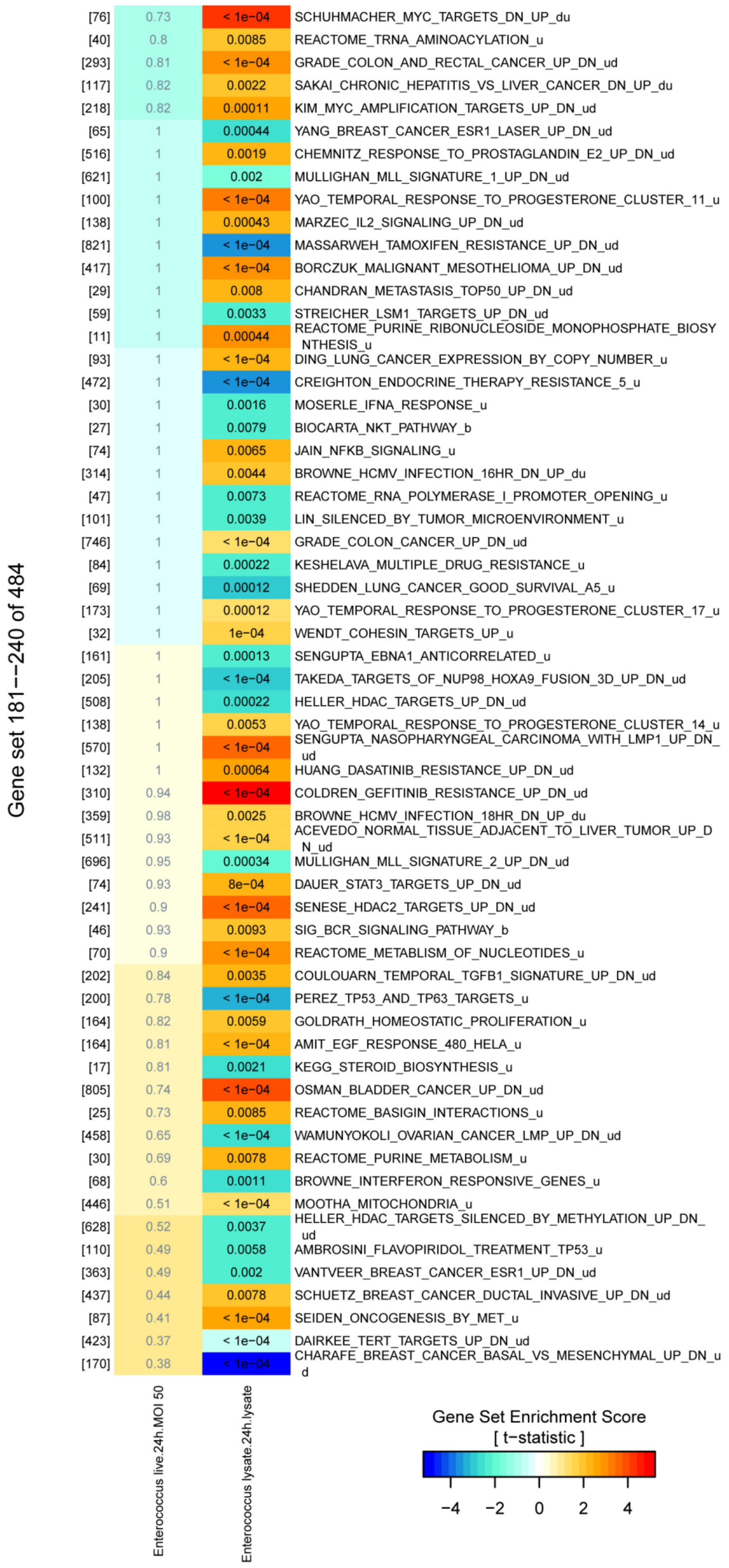

Gene set 181--240 of 484
[76] 0.73 < 1e-04 SCHUHMACHER_MYC_TARGETS_DN_UP_du
[40] 0.8 0.0085 REACTOME_TRNA_AMINOACYLATION_u
[293] 0.81 < 1e-04 GRADE_COLON_AND_RECTAL_CANCER_UP_DN_ud
[117] 0.82 0.0022 SAKAI_CHRONIC_HEPATITIS_VS_LIVER_CANCER_DN_UP_du
[218] 0.82 0.00011 KIM_MYC_AMPLIFICATION_TARGETS_UP_DN_ud
[65] 1 0.00044 YANG_BREAST_CANCER_ESR1_LASER_UP_DN_ud
[516] 1 0.0019 CHEMNITZ_RESPONSE_TO_PROSTAGLANDIN_E2_UP_DN_ud
[621] 1 0.002 MULLIGHAN_MLL_SIGNATURE_1_UP_DN_ud
[100] 1 < 1e-04 YAO_TEMPORAL_RESPONSE_TO_PROGESTERONE_CLUSTER_11_u
[138] 1 0.00043 MARZEC_IL2_SIGNALING_UP_DN_ud
[821] 1 < 1e-04 MASSARWEH_TAMOXIFEN_RESISTANCE_UP_DN_ud
[417] 1 < 1e-04 BORCZUK_MALIGNANT_MESOTHELIOMA_UP_DN_ud
[29] 1 0.008 CHANDRAN_METASTASIS_TOP50_UP_DN_ud
[59] 1 0.0033 STREICHER_LSM1_TARGETS_UP_DN_ud
[11] 1 0.00044 REACTOME_PURINE_RIBONUCLEOSIDE_MONOPHOSPHATE_BIOSY NTHESIS_u
[93] 1 < 1e-04 DING_LUNG_CANCER_EXPRESSION_BY_COPY_NUMBER_u
[472] 1 < 1e-04 CREIGHTON_ENDOCRINE_THERAPY_RESISTANCE_5_u
[30] 1 0.0016 MOSERLE_IFNA_RESPONSE_u
[27] 1 0.0079 BIOCARTA_NKT_PATHWAY_b
[74] 1 0.0065 JAIN_NFKB_SIGNALING_u
[314] 1 0.0044 BROWNE_HCMV_INFECTION_16HR_DN_UP_du
[47] 1 0.0073 REACTOME_RNA_POLYMERASE_I_PROMOTER_OPENING_u
[101] 1 0.0039 LIN_SILENCED_BY_TUMOR_MICROENVIRONMENT_u
[746] 1 < 1e-04 GRADE_COLON_CANCER_UP_DN_ud
[84] 1 0.00022 KESHELAVA_MULTIPLE_DRUG_RESISTANCE_u
[69] 1 0.00012 SHEDDEN_LUNG_CANCER_GOOD_SURVIVAL_A5_u
[173] 1 0.00012 YAO_TEMPORAL_RESPONSE_TO_PROGESTERONE_CLUSTER_17_u
[32] 1 1e-04 WENDT_COHESIN_TARGETS_UP_u
[161] 1 0.00013 SENGUPTA_EBNA1_ANTICORRELATED_u
[205] 1 < 1e-04 TAKEDA_TARGETS_OF_NUP98_HOXA9_FUSION_3D_UP_DN_ud
[508] 1 0.00022 HELLER_HDAC_TARGETS_UP_DN_ud
[138] 1 0.0053 YAO_TEMPORAL_RESPONSE_TO_PROGESTERONE_CLUSTER_14_u
[570] 1 < 1e-04 SENGUPTA_NASOPHARYNGEAL_CARCINOMA_WITH_LMP1_UP_DN_ ud
[132] 1 0.00064 HUANG_DASATINIB_RESISTANCE_UP_DN_ud
[310] 0.94 < 1e-04 COLDREN_GEFITINIB_RESISTANCE_UP_DN_ud
[359] 0.98 0.0025 BROWNE_HCMV_INFECTION_18HR_DN_UP_du
[511] 0.93 < 1e-04 ACEVEDO_NORMAL_TISSUE_ADJACENT_TO_LIVER_TUMOR_UP_D N_ud
[696] 0.95 0.00034 MULLIGHAN_MLL_SIGNATURE_2_UP_DN_ud
[74] 0.93 8e-04 DAUER_STAT3_TARGETS_UP_DN_ud
[241] 0.9 < 1e-04 SENESE_HDAC2_TARGETS_UP_DN_ud
[46] 0.93 0.0093 SIG_BCR_SIGNALING_PATHWAY_b
[70] 0.9 < 1e-04 REACTOME_METABLISM_OF_NUCLEOTIDES_u
[202] 0.84 0.0035 COULOUARN_TEMPORAL_TGFB1_SIGNATURE_UP_DN_ud
[200] 0.78 < 1e-04 PEREZ_TP53_AND_TP63_TARGETS_u
[164] 0.82 0.0059 GOLDRATH_HOMEOSTATIC_PROLIFERATION_u
[164] 0.81 < 1e-04 AMIT_EGF_RESPONSE_480_HELA_u
[17] 0.81 0.0021 KEGG_STEROID_BIOSYNTHESIS_u
[805] 0.74 < 1e-04 OSMAN_BLADDER_CANCER_UP_DN_ud
[25] 0.73 0.0085 REACTOME_BASIGIN_INTERACTIONS_u
[458] 0.65 < 1e-04 WAMUNYOKOLI_OVARIAN_CANCER_LMP_UP_DN_ud
[30] 0.69 0.0078 REACTOME_PURINE_METABOLISM_u
[68] 0.6 0.0011 BROWNE_INTERFERON_RESPONSIVE_GENES_u
[446] 0.51 < 1e-04 MOOTHA_MITOCHONDRIA_u
[628] 0.52 0.0037 HELLER_HDAC_TARGETS_SILENCED_BY_METHYLATION_UP_DN_ ud
[110] 0.49 0.0058 AMBROSINI_FLAVOPIRIDOL_TREATMENT_TP53_u
[363] 0.49 0.002 VANTVEER_BREAST_CANCER_ESR1_UP_DN_ud
[437] 0.44 0.0078 SCHUETZ_BREAST_CANCER_DUCTAL_INVASIVE_UP_DN_ud
[87] 0.41 < 1e-04 SEIDEN_ONCOGENESIS_BY_MET_u
[423] 0.37 < 1e-04 DAIRKEE_TERT_TARGETS_UP_DN_ud
[170] 0.38 < 1e-04 CHARAFE_BREAST_CANCER_BASAL_VS_MESENCHYMAL_UP_DN_u d
Enterococcus live.24h.MOI 50
Enterococcus lysate.24h.lysate
Gene Set Enrichment Score
[t−statistic]
−4
−2
0
2
4

Gene set 241--300 of 484

	Enterococcus live.24h.MOI 50	Enterococcus lysate.24h.lysate	
[25]	0.39	< 1e−04	KOBAYASHI_EGFR_SIGNALING_6HR_UP_DN_ud
[36]	0.39	0.0092	BIOCARTA_MET_PATHWAY_b
[695]	0.36	0.00022	DEURIG_T_CELL_PROLYMPHOCYTIC_LEUKEMIA_UP_DN_ud
[621]	0.31	< 1e−04	PARENT_MTOR_SIGNALING_UP_DN_ud
[99]	0.36	< 1e−04	JI_RESPONSE_TO_FSH_DN_UP_du
[180]	0.28	0.0074	KEGG_ENDOCYTOSIS_u
[428]	0.22	< 1e−04	MOOTHA_HUMAN_MITODB_6_2002_u
[701]	0.22	< 1e−04	SENESE_HDAC1_TARGETS_UP_DN_ud
[158]	0.24	0.00096	SMID_BREAST_CANCER_ERBB2_UP_DN_ud
[347]	0.18	< 1e−04	PEREZ_TP63_TARGETS_u
[326]	0.2	0.0059	FARMER_BREAST_CANCER_APOCRINE VS LUMINAL_u
[213]	0.2	0.0013	REACTOME_METABOLISM_OF_PROTEINS_u
[43]	0.18	0.0075	AMIT_EGF_RESPONSE_480_MCF10A_u
[86]	0.15	0.006	BIOCARTA_MAPK_PATHWAY_b
[250]	0.13	0.0017	LEE_LIVER_CANCER_SURVIVAL_DN_UP_du
[712]	0.092	< 1e−04	CREIGHTON_ENDOCRINE_THERAPY_RESISTANCE_3_u
[35]	0.13	0.0076	NIKOLSKY_BREAST_CANCER_1Q21_AMPLICON_u
[237]	0.09	0.001	NAGASHIMA_NRG1_SIGNALING_UP_DN_ud
[861]	0.089	< 1e−04	LOPEZ_MBD_TARGETS_u
[511]	0.098	< 1e−04	WANG_SMARCE1_TARGETS_DN_UP_du
[898]	0.063	< 1e−04	CHARAFE_BREAST_CANCER_LUMINAL_VS_MESENCHYMAL_UP_DN_ud
[294]	0.067	0.0021	WELCSH_BRCA1_TARGETS_1_UP_DN_ud
[109]	0.071	0.00012	DE_YY1_TARGETS_UP_DN_ud
[11]	0.065	0.0027	GRANDVAUX_IFN_RESPONSE_NOT_VIA_IRF3_u
[346]	0.042	0.00012	HUTTMANN_B_CLL_POOR_SURVIVAL_UP_DN_ud
[238]	0.049	< 1e−04	IZADPANAH_STEM_CELL_ADIPOSE_VS_BONE_UP_DN_ud
[83]	0.049	0.0085	KEGG_TGF_BETA_SIGNALING_PATHWAY_b
[188]	0.048	< 1e−04	LIEN_BREAST_CARCINOMA_METAPLASTIC_VS_DUCTAL_UP_DN_ud
[324]	0.04	< 1e−04	KEGG_PATHWAYS_IN_CANCER_b
[27]	0.027	< 1e−04	EINAV_INTERFERON_SIGNATURE_IN_CANCER_u
[14]	0.03	0.0019	BOWIE_RESPONSE_TO_TAMOXIFEN_u
[88]	0.02	0.003	BROWNE_HCMV_INFECTION_2HR_UP_DN_ud
[114]	0.018	0.0049	KEGG_OXIDATIVE_PHOSPHORYLATION_u
[81]	0.017	0.0014	WANG_HCP_PROSTATE_CANCER_u
[19]	0.019	0.00069	TSAI_DNAJB4_TARGETS_UP_DN_ud
[150]	0.014	0.0055	VANTVEER_BREAST_CANCER_METASTASIS_DN_UP_du
[440]	0.0086	0.061	RHEIN_ALL_GLUCOCORTICOID_THERAPY_UP_DN_ud
[152]	0.0099	0.00046	FERRANDO_T_ALL_WITH_MLL_ENL_FUSION_UP_DN_ud
[75]	0.0097	0.15	ZHANG_RESPONSE_TO_CANTHARIDIN_DN_UP_du
[39]	0.0089	0.26	SESTO_RESPONSE_TO_UV_C6_u
[32]	0.0098	0.26	HOUSTIS_ROS_u
[127]	0.0083	0.25	KEGG_NATURAL_KILLER_CELL_MEDIATED_CYTOTOXICITY_u
[33]	0.0092	0.57	STEARMAN_TUMOR_FIELD_EFFECT_UP_u
[420]	0.0064	0.12	PASQUALUCCI_LYMPHOMA_BY_GC_STAGE_UP_DN_ud
[30]	0.0099	1	VALK_AML_CLUSTER_1_u
[114]	0.0064	0.0013	NIKOLSKY_BREAST_CANCER_16P13_AMPLICON_u
[595]	0.0056	< 1e−04	FLECHNER_BIOPSY_KIDNEY_TRANSPLANT_OK_VS_DONOR_DN_UP_du
[47]	0.0086	0.89	KEGG_AUTOIMMUNE_THYROID_DISEASE_u
[184]	0.0063	0.4	WU_CELL_MIGRATION_u
[76]	0.007	0.35	REACTOME_MEMBRANE_TRAFFICKING_u
[156]	0.0065	0.016	UEDA_PERIFERAL_CLOCK_u
[120]	0.0063	0.0052	KEGG_LYSOSOME_u
[30]	0.0071	0.018	KANG_CISPLATIN_RESISTANCE_UP_DN_ud
[74]	0.0061	0.16	SESTO_RESPONSE_TO_UV_C7_u
[15]	0.0098	0.082	KEGG_OTHER_GLYCAN_DEGRADATION_u
[17]	0.0088	0.57	REACTOME_FANCONI_ANEMIA_PATHWAY_b
[63]	0.0056	0.021	REACTOME_ELECTRON_TRANSPORT_CHAIN_u
[22]	0.0077	1	ABE_INNER_EAR_u
[52]	0.0056	0.43	KEGG_ARGININE_AND_PROLINE_METABOLISM_u
[172]	0.0043	0.00047	LIU_CMYB_TARGETS_UP_DN_ud

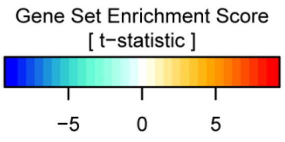

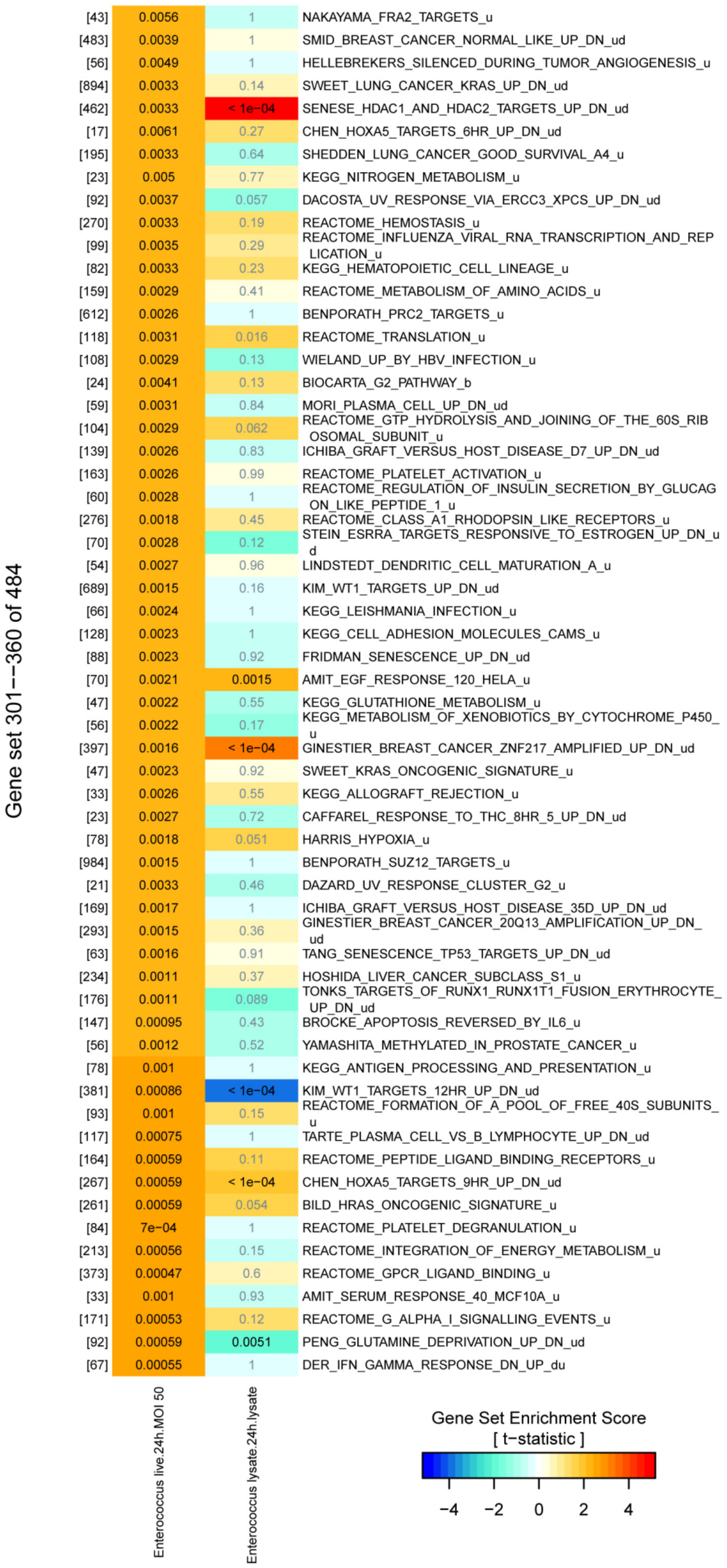
Gene set 301--360 of 484
[43] 0.0056 1 NAKAYAMA_FRA2_TARGETS_u
[483] 0.0039 1 SMID_BREAST_CANCER_NORMAL_LIKE_UP_DN_ud
[56] 0.0049 1 HELLEBREKERS_SILENCED_DURING_TUMOR_ANGIOGENESIS_u
[894] 0.0033 0.14 SWEET_LUNG_CANCER_KRAS_UP_DN_ud
[462] 0.0033 < 1e−04 SENESE_HDAC1_AND_HDAC2_TARGETS_UP_DN_ud
[17] 0.0061 0.27 CHEN_HOXA5_TARGETS_6HR_UP_DN_ud
[195] 0.0033 0.64 SHEDDEN_LUNG_CANCER_GOOD_SURVIVAL_A4_u
[23] 0.005 0.77 KEGG_NITROGEN_METABOLISM_u
[92] 0.0037 0.057 DACOSTA_UV_RESPONSE_VIA_ERCC3_XPCS_UP_DN_ud
[270] 0.0033 0.19 REACTOME_HEMOSTASIS_u
[99] 0.0035 0.29 REACTOME_INFLUENZA_VIRAL_RNA_TRANSCRIPTION_AND_REPLICATION_u
[82] 0.0033 0.23 KEGG_HEMATOPOIETIC_CELL_LINEAGE_u
[159] 0.0029 0.41 REACTOME_METABOLISM_OF_AMINO_ACIDS_u
[612] 0.0026 1 BENPORATH_PRC2_TARGETS_u
[118] 0.0031 0.016 REACTOME_TRANSLATION_u
[108] 0.0029 0.13 WIELAND_UP_BY_HBV_INFECTION_u
[24] 0.0041 0.13 BIOCARTA_G2_PATHWAY_b
[59] 0.0031 0.84 MORI_PLASMA_CELL_UP_DN_ud
[104] 0.0029 0.062 REACTOME_GTP_HYDROLYSIS_AND_JOINING_OF_THE_60S_RIBOSOMAL_SUBUNIT_u
[139] 0.0026 0.83 ICHIBA_GRAFT_VERSUS_HOST_DISEASE_D7_UP_DN_ud
[163] 0.0026 0.99 REACTOME_PLATELET_ACTIVATION_u
[60] 0.0028 1 REACTOME_REGULATION_OF_INSULIN_SECRETION_BY_GLUCAGON_LIKE_PEPTIDE_1_u
[276] 0.0018 0.45 REACTOME_CLASS_A1_RHODOPSIN_LIKE_RECEPTORS_u
[70] 0.0028 0.12 STEIN_ESRRA_TARGETS_RESPONSIVE_TO_ESTROGEN_UP_DN_ud
[54] 0.0027 0.96 LINDSTEDT_DENDRITIC_CELL_MATURATION_A_u
[689] 0.0015 0.16 KIM_WT1_TARGETS_UP_DN_ud
[66] 0.0024 1 KEGG_LEISHMANIA_INFECTION_u
[128] 0.0023 1 KEGG_CELL_ADHESION_MOLECULES_CAMS_u
[88] 0.0023 0.92 FRIDMAN_SENESCENCE_UP_DN_ud
[70] 0.0021 0.0015 AMIT_EGF_RESPONSE_120_HELA_u
[47] 0.0022 0.55 KEGG_GLUTATHIONE_METABOLISM_u
[56] 0.0022 0.17 KEGG_METABOLISM_OF_XENOBIOTICS_BY_CYTOCHROME_P450_u
[397] 0.0016 < 1e−04 GINESTIER_BREAST_CANCER_ZNF217_AMPLIFIED_UP_DN_ud
[47] 0.0023 0.92 SWEET_KRAS_ONCOGENIC_SIGNATURE_u
[33] 0.0026 0.55 KEGG_ALLOGRAFT_REJECTION_u
[23] 0.0027 0.72 CAFFAREL_RESPONSE_TO_THC_8HR_5_UP_DN_ud
[78] 0.0018 0.051 HARRIS_HYPOXIA_u
[984] 0.0015 1 BENPORATH_SUZ12_TARGETS_u
[21] 0.0033 0.46 DAZARD_UV_RESPONSE_CLUSTER_G2_u
[169] 0.0017 1 ICHIBA_GRAFT_VERSUS_HOST_DISEASE_35D_UP_DN_ud
[293] 0.0015 0.36 GINESTIER_BREAST_CANCER_20Q13_AMPLIFICATION_UP_DN_ud
[63] 0.0016 0.91 TANG_SENESCENCE_TP53_TARGETS_UP_DN_ud
[234] 0.0011 0.37 HOSHIDA_LIVER_CANCER_SUBCLASS_S1_u
[176] 0.0011 0.089 TONKS_TARGETS_OF_RUNX1_RUNX1T1_FUSION_ERYTHROCYTE_UP_DN_ud
[147] 0.00095 0.43 BROCKE_APOPTOSIS_REVERSED_BY_IL6_u
[56] 0.0012 0.52 YAMASHITA_METHYLATED_IN_PROSTATE_CANCER_u
[78] 0.001 1 KEGG_ANTIGEN_PROCESSING_AND_PRESENTATION_u
[381] 0.00086 < 1e−04 KIM_WT1_TARGETS_12HR_UP_DN_ud
[93] 0.001 0.15 REACTOME_FORMATION_OF_A_POOL_OF_FREE_40S_SUBUNITS_u
[117] 0.00075 1 TARTE_PLASMA_CELL_VS_B_LYMPHOCYTE_UP_DN_ud
[164] 0.00059 0.11 REACTOME_PEPTIDE_LIGAND_BINDING_RECEPTORS_u
[267] 0.00059 < 1e−04 CHEN_HOXA5_TARGETS_9HR_UP_DN_ud
[261] 0.00059 0.054 BILD_HRAS_ONCOGENIC_SIGNATURE_u
[84] 7e−04 1 REACTOME_PLATELET_DEGRANULATION_u
[213] 0.00056 0.15 REACTOME_INTEGRATION_OF_ENERGY_METABOLISM_u
[373] 0.00047 0.6 REACTOME_GPCR_LIGAND_BINDING_u
[33] 0.001 0.93 AMIT_SERUM_RESPONSE_40_MCF10A_u
[171] 0.00053 0.12 REACTOME_G_ALPHA_I_SIGNALLING_EVENTS_u
[92] 0.00059 0.0051 PENG_GLUTAMINE_DEPRIVATION_UP_DN_ud
[67] 0.00055 1 DER_IFN_GAMMA_RESPONSE_DN_UP_du
Enterococcus live.24h.MOI 50
Enterococcus lysate.24h.lysate
Gene Set Enrichment Score
[t−statistic]
−4 −2 0 2 4

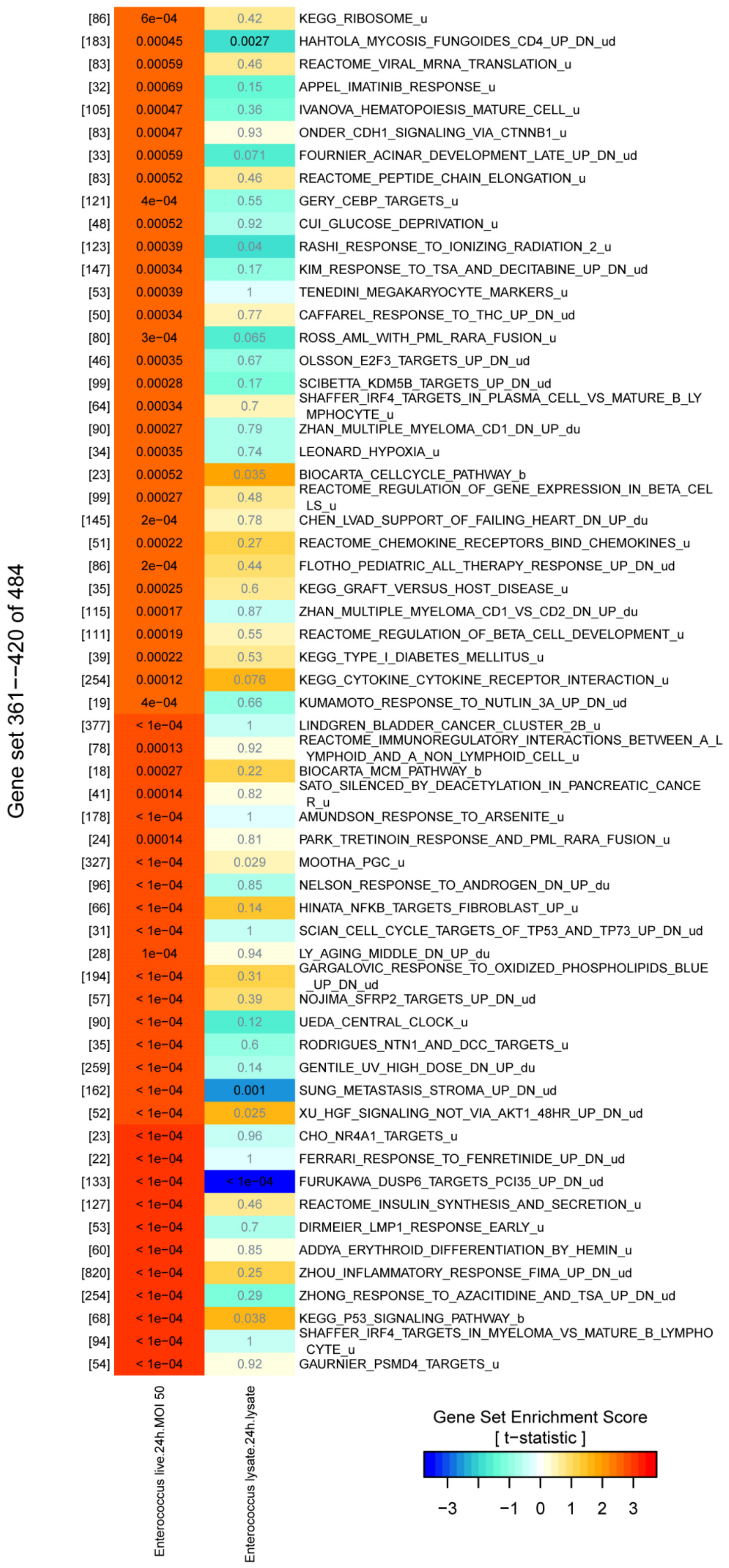
Gene set 361−−420 of 484
[86] 6e−04 0.42 KEGG_RIBOSOME_u
[183] 0.00045 0.0027 HAHTOLA_MYCOSIS_FUNGOIDES_CD4_UP_DN_ud
[83] 0.00059 0.46 REACTOME_VIRAL_MRNA_TRANSLATION_u
[32] 0.00069 0.15 APPEL_IMATINIB_RESPONSE_u
[105] 0.00047 0.36 IVANOVA_HEMATOPOIESIS_MATURE_CELL_u
[83] 0.00047 0.93 ONDER_CDH1_SIGNALING_VIA_CTNNB1_u
[33] 0.00059 0.071 FOURNIER_ACINAR_DEVELOPMENT_LATE_UP_DN_ud
[83] 0.00052 0.46 REACTOME_PEPTIDE_CHAIN_ELONGATION_u
[121] 4e−04 0.55 GERY_CEBP_TARGETS_u
[48] 0.00052 0.92 CUI_GLUCOSE_DEPRIVATION_u
[123] 0.00039 0.04 RASHI_RESPONSE_TO_IONIZING_RADIATION_2_u
[147] 0.00034 0.17 KIM_RESPONSE_TO_TSA_AND_DECITABINE_UP_DN_ud
[53] 0.00039 1 TENEDINI_MEGAKARYOCYTE_MARKERS_u
[50] 0.00034 0.77 CAFFAREL_RESPONSE_TO_THC_UP_DN_ud
[80] 3e−04 0.065 ROSS_AML_WITH_PML_RARA_FUSION_u
[46] 0.00035 0.67 OLSSON_E2F3_TARGETS_UP_DN_ud
[99] 0.00028 0.17 SCIBETTA_KDM5B_TARGETS_UP_DN_ud
[64] 0.00034 0.7 SHAFFER_IRF4_TARGETS_IN_PLASMA_CELL_VS_MATURE_B_LY MPHOCYTE_u
[90] 0.00027 0.79 ZHAN_MULTIPLE_MYELOMA_CD1_DN_UP_du
[34] 0.00035 0.74 LEONARD_HYPOXIA_u
[23] 0.00052 0.035 BIOCARTA_CELLCYCLE_PATHWAY_b
[99] 0.00027 0.48 REACTOME_REGULATION_OF_GENE_EXPRESSION_IN_BETA_CEL LS_u
[145] 2e−04 0.78 CHEN_LVAD_SUPPORT_OF_FAILING_HEART_DN_UP_du
[51] 0.00022 0.27 REACTOME_CHEMOKINE_RECEPTORS_BIND_CHEMOKINES_u
[86] 2e−04 0.44 FLOTHO_PEDIATRIC_ALL_THERAPY_RESPONSE_UP_DN_ud
[35] 0.00025 0.6 KEGG_GRAFT_VERSUS_HOST_DISEASE_u
[115] 0.00017 0.87 ZHAN_MULTIPLE_MYELOMA_CD1_VS_CD2_DN_UP_du
[111] 0.00019 0.55 REACTOME_REGULATION_OF_BETA_CELL_DEVELOPMENT_u
[39] 0.00022 0.53 KEGG_TYPE_I_DIABETES_MELLITUS_u
[254] 0.00012 0.076 KEGG_CYTOKINE_CYTOKINE_RECEPTOR_INTERACTION_u
[19] 4e−04 0.66 KUMAMOTO_RESPONSE_TO_NUTLIN_3A_UP_DN_ud
[377] < 1e−04 1 LINDGREN_BLADDER_CANCER_CLUSTER_2B_u
[78] 0.00013 0.92 REACTOME_IMMUNOREGULATORY_INTERACTIONS_BETWEEN_A_L YMPHOID_AND_A_NON_LYMPHOID_CELL_u
[18] 0.00027 0.22 BIOCARTA_MCM_PATHWAY_b
[41] 0.00014 0.82 SATO_SILENCED_BY_DEACETYLATION_IN_PANCREATIC_CANCE R_u
[178] < 1e−04 1 AMUNDSON_RESPONSE_TO_ARSENITE_u
[24] 0.00014 0.81 PARK_TRETINOIN_RESPONSE_AND_PML_RARA_FUSION_u
[327] < 1e−04 0.029 MOOTHA_PGC_u
[96] < 1e−04 0.85 NELSON_RESPONSE_TO_ANDROGEN_DN_UP_du
[66] < 1e−04 0.14 HINATA_NFKB_TARGETS_FIBROBLAST_UP_u
[31] < 1e−04 1 SCIAN_CELL_CYCLE_TARGETS_OF_TP53_AND_TP73_UP_DN_ud
[28] 1e−04 0.94 LY_AGING_MIDDLE_DN_UP_du
[194] < 1e−04 0.31 GARGALOVIC_RESPONSE_TO_OXIDIZED_PHOSPHOLIPIDS_BLUE _UP_DN_ud
[57] < 1e−04 0.39 NOJIMA_SFRP2_TARGETS_UP_DN_ud
[90] < 1e−04 0.12 UEDA_CENTRAL_CLOCK_u
[35] < 1e−04 0.6 RODRIGUES_NTN1_AND_DCC_TARGETS_u
[259] < 1e−04 0.14 GENTILE_UV_HIGH_DOSE_DN_UP_du
[162] < 1e−04 0.001 SUNG_METASTASIS_STROMA_UP_DN_ud
[52] < 1e−04 0.025 XU_HGF_SIGNALING_NOT_VIA_AKT1_48HR_UP_DN_ud
[23] < 1e−04 0.96 CHO_NR4A1_TARGETS_u
[22] < 1e−04 1 FERRARI_RESPONSE_TO_FENRETINIDE_UP_DN_ud
[133] < 1e−04 < 1e−04 FURUKAWA_DUSP6_TARGETS_PCI35_UP_DN_ud
[127] < 1e−04 0.46 REACTOME_INSULIN_SYNTHESIS_AND_SECRETION_u
[53] < 1e−04 0.7 DIRMEIER_LMP1_RESPONSE_EARLY_u
[60] < 1e−04 0.85 ADDYA_ERYTHROID_DIFFERENTIATION_BY_HEMIN_u
[820] < 1e−04 0.25 ZHOU_INFLAMMATORY_RESPONSE_FIMA_UP_DN_ud
[254] < 1e−04 0.29 ZHONG_RESPONSE_TO_AZACITIDINE_AND_TSA_UP_DN_ud
[68] < 1e−04 0.038 KEGG_P53_SIGNALING_PATHWAY_b
[94] < 1e−04 1 SHAFFER_IRF4_TARGETS_IN_MYELOMA_VS_MATURE_B_LYMPHO CYTE_u
[54] < 1e−04 0.92 GAURNIER_PSMD4_TARGETS_u
Enterococcus live.24h.MOI 50
Enterococcus lysate.24h.lysate
Gene Set Enrichment Score
[t−statistic]
−3 −1 0 1 2 3

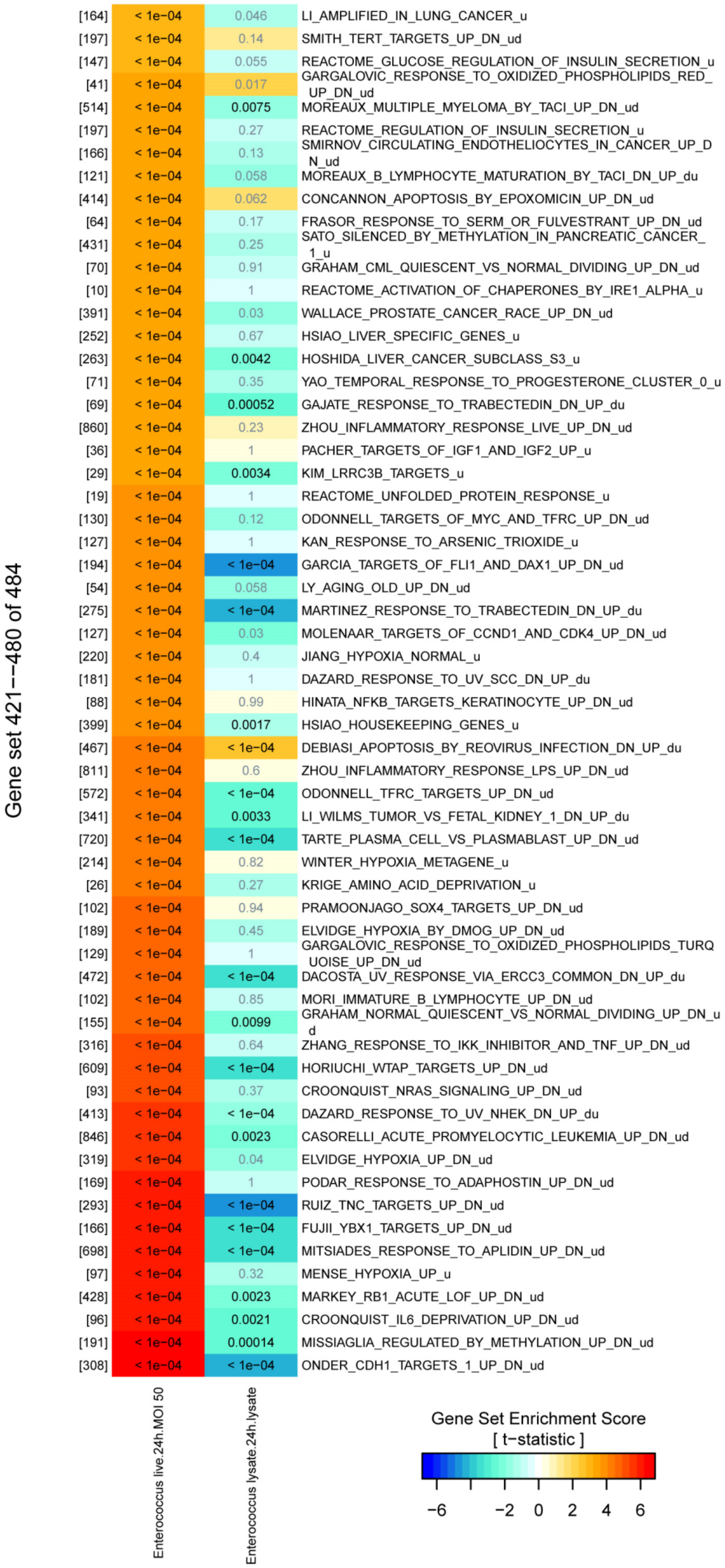
Gene set 421--480 of 484
[164] < 1e-04 0.046 LI_AMPLIFIED_IN_LUNG_CANCER_u
[197] < 1e-04 0.14 SMITH_TERT_TARGETS_UP_DN_ud
[147] < 1e-04 0.055 REACTOME_GLUCOSE_REGULATION_OF_INSULIN_SECRETION_u
[41] < 1e-04 0.017 GARGALOVIC_RESPONSE_TO_OXIDIZED_PHOSPHOLIPIDS_RED_UP_DN_ud
[514] < 1e-04 0.0075 MOREAUX_MULTIPLE_MYELOMA_BY_TACI_UP_DN_ud
[197] < 1e-04 0.27 REACTOME_REGULATION_OF_INSULIN_SECRETION_u
[166] < 1e-04 0.13 SMIRNOV_CIRCULATING_ENDOTHELIOCYTES_IN_CANCER_UP_DN_ud
[121] < 1e-04 0.058 MOREAUX_B_LYMPHOCYTE_MATURATION_BY_TACI_DN_UP_du
[414] < 1e-04 0.062 CONCANNON_APOPTOSIS_BY_EPOXOMICIN_UP_DN_ud
[64] < 1e-04 0.17 FRASOR_RESPONSE_TO_SERM_OR_FULVESTRANT_UP_DN_ud
[431] < 1e-04 0.25 SATO_SILENCED_BY_METHYLATION_IN_PANCREATIC_CANCER_1_u
[70] < 1e-04 0.91 GRAHAM_CML_QUIESCENT_VS_NORMAL_DIVIDING_UP_DN_ud
[10] < 1e-04 1 REACTOME_ACTIVATION_OF_CHAPERONES_BY_IRE1_ALPHA_u
[391] < 1e-04 0.03 WALLACE_PROSTATE_CANCER_RACE_UP_DN_ud
[252] < 1e-04 0.67 HSIAO_LIVER_SPECIFIC_GENES_u
[263] < 1e-04 0.0042 HOSHIDA_LIVER_CANCER_SUBCLASS_S3_u
[71] < 1e-04 0.35 YAO_TEMPORAL_RESPONSE_TO_PROGESTERONE_CLUSTER_0_u
[69] < 1e-04 0.00052 GAJATE_RESPONSE_TO_TRABECTEDIN_DN_UP_du
[860] < 1e-04 0.23 ZHOU_INFLAMMATORY_RESPONSE_LIVE_UP_DN_ud
[36] < 1e-04 1 PACHER_TARGETS_OF_IGF1_AND_IGF2_UP_u
[29] < 1e-04 0.0034 KIM_LRRC3B_TARGETS_u
[19] < 1e-04 1 REACTOME_UNFOLDED_PROTEIN_RESPONSE_u
[130] < 1e-04 0.12 ODONNELL_TARGETS_OF_MYC_AND_TFRC_UP_DN_ud
[127] < 1e-04 1 KAN_RESPONSE_TO_ARSENIC_TRIOXIDE_u
[194] < 1e-04 < 1e-04 GARCIA_TARGETS_OF_FLI1_AND_DAX1_UP_DN_ud
[54] < 1e-04 0.058 LY_AGING_OLD_UP_DN_ud
[275] < 1e-04 < 1e-04 MARTINEZ_RESPONSE_TO_TRABECTEDIN_DN_UP_du
[127] < 1e-04 0.03 MOLENAAR_TARGETS_OF_CCND1_AND_CDK4_UP_DN_ud
[220] < 1e-04 0.4 JIANG_HYPOXIA_NORMAL_u
[181] < 1e-04 1 DAZARD_RESPONSE_TO_UV_SCC_DN_UP_du
[88] < 1e-04 0.99 HINATA_NFKB_TARGETS_KERATINOCYTE_UP_DN_ud
[399] < 1e-04 0.0017 HSIAO_HOUSEKEEPING_GENES_u
[467] < 1e-04 < 1e-04 DEBIASI_APOPTOSIS_BY_REOVIRUS_INFECTION_DN_UP_du
[811] < 1e-04 0.6 ZHOU_INFLAMMATORY_RESPONSE_LPS_UP_DN_ud
[572] < 1e-04 < 1e-04 ODONNELL_TFRC_TARGETS_UP_DN_ud
[341] < 1e-04 0.0033 LI_WILMS_TUMOR_VS_FETAL_KIDNEY_1_DN_UP_du
[720] < 1e-04 < 1e-04 TARTE_PLASMA_CELL_VS_PLASMABLAST_UP_DN_ud
[214] < 1e-04 0.82 WINTER_HYPOXIA_METAGENE_u
[26] < 1e-04 0.27 KRIGE_AMINO_ACID_DEPRIVATION_u
[102] < 1e-04 0.94 PRAMOONJAGO_SOX4_TARGETS_UP_DN_ud
[189] < 1e-04 0.45 ELVIDGE_HYPOXIA_BY_DMOG_UP_DN_ud
[129] < 1e-04 1 GARGALOVIC_RESPONSE_TO_OXIDIZED_PHOSPHOLIPIDS_TURQUOISE_UP_DN_ud
[472] < 1e-04 < 1e-04 DACOSTA_UV_RESPONSE_VIA_ERCC3_COMMON_DN_UP_du
[102] < 1e-04 0.85 MORI_IMMATURE_B_LYMPHOCYTE_UP_DN_ud
[155] < 1e-04 0.0099 GRAHAM_NORMAL_QUIESCENT_VS_NORMAL_DIVIDING_UP_DN_ud
[316] < 1e-04 0.64 ZHANG_RESPONSE_TO_IKK_INHIBITOR_AND_TNF_UP_DN_ud
[609] < 1e-04 < 1e-04 HORIUCHI_WTAP_TARGETS_UP_DN_ud
[93] < 1e-04 0.37 CROONQUIST_NRAS_SIGNALING_UP_DN_ud
[413] < 1e-04 < 1e-04 DAZARD_RESPONSE_TO_UV_NHEK_DN_UP_du
[846] < 1e-04 0.0023 CASORELLI_ACUTE_PROMYELOCYTIC_LEUKEMIA_UP_DN_ud
[319] < 1e-04 0.04 ELVIDGE_HYPOXIA_UP_DN_ud
[169] < 1e-04 1 PODAR_RESPONSE_TO_ADAPHOSTIN_UP_DN_ud
[293] < 1e-04 < 1e-04 RUIZ_TNC_TARGETS_UP_DN_ud
[166] < 1e-04 < 1e-04 FUJII_YBX1_TARGETS_UP_DN_ud
[698] < 1e-04 < 1e-04 MITSIADES_RESPONSE_TO_APLIDIN_UP_DN_ud
[97] < 1e-04 0.32 MENSE_HYPOXIA_UP_u
[428] < 1e-04 0.0023 MARKEY_RB1_ACUTE_LOF_UP_DN_ud
[96] < 1e-04 0.0021 CROONQUIST_IL6_DEPRIVATION_UP_DN_ud
[191] < 1e-04 0.00014 MISSIAGLIA_REGULATED_BY_METHYLATION_UP_DN_ud
[308] < 1e-04 < 1e-04 ONDER_CDH1_TARGETS_1_UP_DN_ud
Enterococcus live.24h.MOI 50
Enterococcus lysate.24h.lysate
Gene Set Enrichment Score
[t−statistic]
−6 −2 0 2 4 6

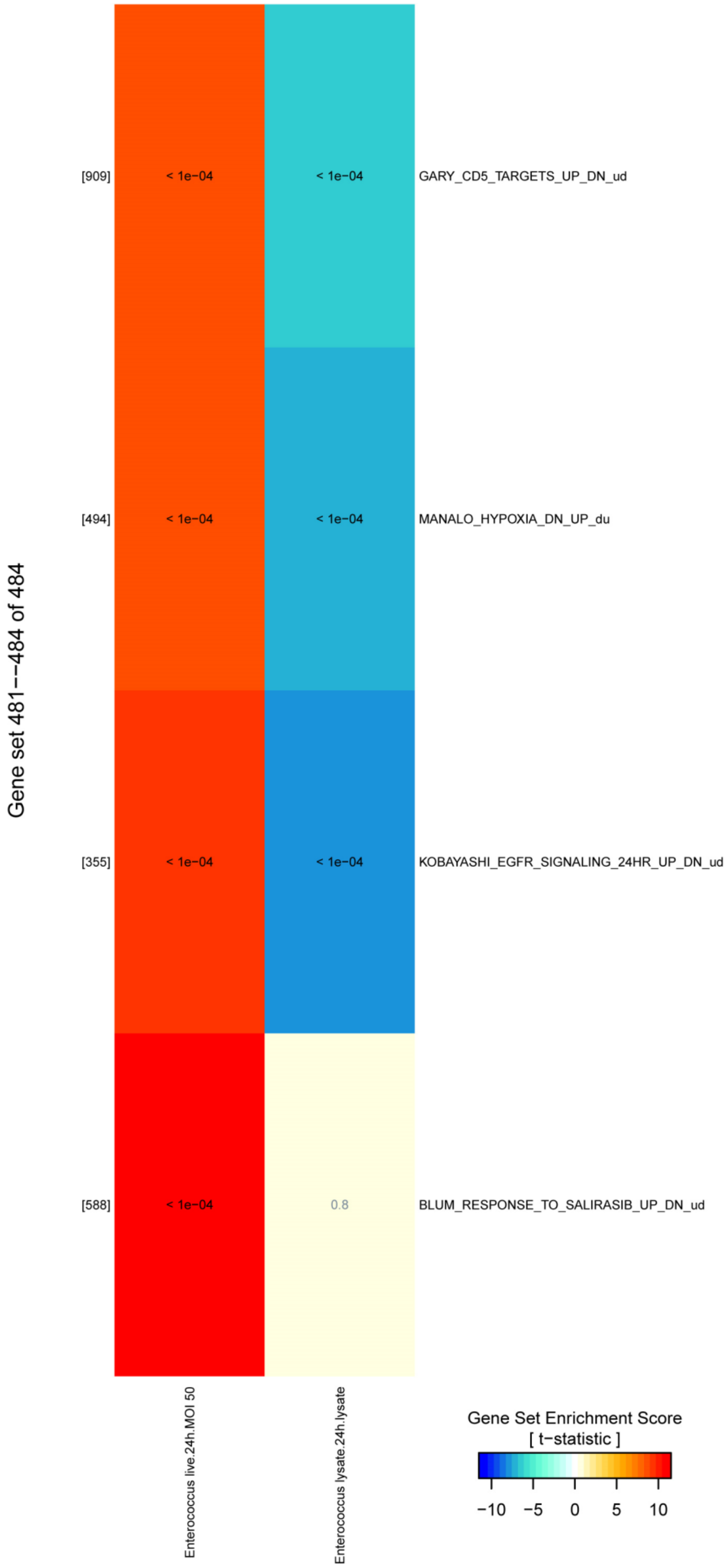

Gene set 481--484 of 484
[909]
< 1e−04
< 1e−04
GARY_CD5_TARGETS_UP_DN_ud
[494]
< 1e−04
< 1e−04
MANALO_HYPOXIA_DN_UP_du
[355]
< 1e−04
< 1e−04
KOBAYASHI_EGFR_SIGNALING_24HR_UP_DN_ud
[588]
< 1e−04
0.8
BLUM_RESPONSE_TO_SALIRASIB_UP_DN_ud
Enterococcus live.24h.MOI 50
Enterococcus lysate.24h.lysate
Gene Set Enrichment Score
[t−statistic]
−10
−5
0
5
10
