# Supplementary material for: Enterococcus faecalis Infection Causes Inflammation, Intracellular Oxphos-Independent ROS Production, and DNA Damage in Human Gastric Cancer Cells
Source: PLoS One. 2013 Apr 30;8(4):e63147. doi: 10.1371/journal.pone.0063147 (PMC3639970; doi:10.1371/journal.pone.0063147)
Supplement: Table S1 — Primers. (DOCX) [file pone.0063147.s003.docx]

**Table S1**

**Primers**

| Primer Name | Sequence 5’→3’ Sequence | Tm (°C) | Amplicon length (bp) |
| --- | --- | --- | --- |
| Mitochondrial D-loop primers | | | |
| C6-CA5- F | accctatgtcgcagtatctgt | 52 |  |
| C6-CA5- R | cagtgtattgctttgaggaggt | 52 |  |
| VectorD-loop-F | caggaaacagctatgaccat | 49 |  |
| VectorD-loop-R | gtaatacgactcactatagggc | 50 |  |
| qPCR primers | | | |
| GAPDH-F | cgaccactttgtcaagctca | 59 | 112 |
| GAPDH-R | ggtggtccaggggtcttact | 60 |  |
| IL-8-F | gcagagggttgtggagaagt | 59 | 149 |
| IL-8-R | ccctacaacagacccacaca | 61 |  |
| IRF-1-F | gtggaagttgtgccggaca | 59 | 91 |
| IRF-1-R | catcctcatctgttgtagcttcagag | 63 |  |
| TNF-α-F | ccagggacctctctctaatcagc | 59 | 107 |
| TNF-α-R | ctcagcttgagggtttgctacaa | 61 |  |
| MLH1-F | TCGACCCTCTCAGGCCAGCA | 63 | 106 |
| MLH1-R | GCAGAATGTGTGAGCGCAAGGC | 64 |  |
| PMS1-F | TGCAGAGCCACTGGAAAAGCCA | 62 | 142 |
| PMS1-R | CGCTGTAAGACGAGGATCAGACAGGT | 66 |  |
| PMS2-F | GGGTCATGTGCCGGCCTTCC | 66 | 137 |
| PMS2-R | GGGGTGGTCCATCTCCCCCA | 66 |  |
| MSH3-F | CCCTGTTTGTCACCCATTATCCGCC | 66 | 139 |
| MSH3-R | TCAGGGACTTGTTCTGCTGCG | 62 |  |
| MSH2-F | GCCCGAGTAGGGGCTGGTGA | 66 | 167 |
| MSH2-R | GCCCATGCTAACCCAAATCCATCGT | 65 |  |
| MSH6-F | GGACTGCATGCGGCGACTGT | 60 | 130 |
| MSH6-R | AGGACCATCACCCCCTCGACT | 58 |  |
