# Supplementary material for: Enterococcus faecalis Infection Causes Inflammation, Intracellular Oxphos-Independent ROS Production, and DNA Damage in Human Gastric Cancer Cells
Source: PLoS One. 2013 Apr 30;8(4):e63147. doi: 10.1371/journal.pone.0063147 (PMC3639970; doi:10.1371/journal.pone.0063147)
Supplement: Table S2 — Expressional levels and fold change of the seven NADPH family members in MKN74 cell cultures during 24 hrs and 5 days of infection with E. faecalis . The phagocyte NADPH oxidase (NOX2) has six homologs: NOX1, NOX3-5 and DUOX1-2, constituting the NOX family of NADPH oxidases. (DOCX) [file pone.0063147.s004.docx]

**Table S2**

**Expression level and fold change of the seven NADPH family members in MKN74 cell cultures during 24 hrs and 5 days of infection with *E. faecalis*.** The phagocyte NADPH oxidase (NOX2) has six homologs: NOX1, NOX3-5 and DUOX1-2, constituting the NOX family of NADPH oxidases.

| **Gene Symbol** | **Mean 24 hrs  expression** | **Fold-Change 24 hrs** | **p-value 24hrs** | **Fold-Change 5 day** | **p-value 5 day** | **Gene Title** |
| --- | --- | --- | --- | --- | --- | --- |
| **NOX1** | 58 | -1.2 | 0.01 | -1.0 | 0.70 | NADPH oxidase 1 |
| **CYBB (NOX2)** | 44 | -1.0 | 0.75 | -1.4 | 0.03 | Cytochrome b-245, beta polypeptide |
| **NOX3** | 15 | 1.0 | 0.52 | 1.0 | 0.81 | NADPH oxidase 3 |
| **NOX4** | 14 | 1.0 | 0.41 | 1.1 | 0.39 | NADPH oxidase 4 |
| **NOX5** | 73 | 1.1 | 0.27 | 1.1 | 0.24 | NADPH oxidase, EF-hand calcium binding domain 5 |
| **DUOX1** | 33 | 1.1 | 0.43 | 1.2 | 0.18 | dual oxidase 1 |
| **DUOX2** | 14 | 1.1 | 0.32 | 1.1 | 0.40 | dual oxidase 2 |
