# Supplementary material for: Enterococcus faecalis Infection Causes Inflammation, Intracellular Oxphos-Independent ROS Production, and DNA Damage in Human Gastric Cancer Cells
Source: PLoS One. 2013 Apr 30;8(4):e63147. doi: 10.1371/journal.pone.0063147 (PMC3639970; doi:10.1371/journal.pone.0063147)
Supplement: Table S3 — DNA damage repair genes identified by microarray analysis. Selected genes are shown. The table shows expressional fold change of these genes in MKN74 cells infected with E. faecalis for 24 hours and 5 days. Difference of means = difference in arbitrary expressional level of control cells and infected cells. * = P value <0.05. (DOCX) [file pone.0063147.s005.docx]

**Table S3**

**DNA damage repair genes identified by microarray analysis.** Selected genes are shown. The table shows expressional fold change of these genes in MKN74 cells infected with *E. faecalis* for 24 hours and 5 days. Difference of means = difference in arbitrary expressional level of control cells and infected cells. * = P value < 0.05

| **Gene** | | **NCBI Reference ID** | | **24 h fold change** | | **Difference of means** | | **P value**  **24 h** | **5 day fold change** | | | **Difference of means** | | | **P value**  **5 day** |
| --- | --- | --- | --- | --- | --- | --- | --- | --- | --- | --- | --- | --- | --- | --- | --- |
| **ATM** | | **NM_000051** | | **-2.0*** | | **59** | | **0.034105** | | | **-2.7*** | **81** | | | **0.005683** |
| **ATR** | | **NM_001184** | | **-1.7*** | | **212** | | **0.036194** | | | **-2.2*** | **119** | | | **0.005376** |
| **MSH2** | **NM_000251** | | **-1.4** | | **254** | | **0.165843** | | | **-2.2*** | | | **173** | **0.008652** | |
| **MLH3** | **NM_001040108** | | **-1.6*** | | **66** | | **0.000261** | | | **-1.5*** | | | **46** | **0.00046** | |
| **PMS1** | **NM_000534** | | **-2.0*** | | **320** | | **0.007877** | | | **-2.5*** | | | **165** | **0.002035** | |
| **PMS2** | **NM_000535** | | **-1.6*** | | **77** | | **0.000214** | | | **1.2*** | | | **36** | **0.046038** | |
| **MSH6** | **NM_000179** | | **-1.8*** | | **519** | | **0.006534** | | | **-1.9*** | | | **250** | **0.003056** | |
| **NEIL3** | **NM_018248** | | **-3.1*** | | **243** | | **4.02E-06** | | | **-2.9*** | | | **60** | **5.80E-06** | |
