# Supplementary material for: Enterococcus faecalis Infection Causes Inflammation, Intracellular Oxphos-Independent ROS Production, and DNA Damage in Human Gastric Cancer Cells
Source: PLoS One. 2013 Apr 30;8(4):e63147. doi: 10.1371/journal.pone.0063147 (PMC3639970; doi:10.1371/journal.pone.0063147)
Supplement: File S1 — (DOCX) [file pone.0063147.s006.docx]

#### File S1

Respirometry of MKN74 cells was performed using an XF24 Extracellular Flux Analyzer (Seahorse Bioscience, North Billerica, MA). Cells were plated at a density of 75.000 cells per well in a 24-well XF cell culture microplate and grown O/N in growth media to ensure plating. Half of the plated MKN74 cells were infected with *E. faecalis* at a MOI of 50 for 4, 8 or 24 hours while the other half was left uninfected. After incubation, cells where washed twice and finally resuspended in 600 µl respiratory media (RPMI 1640 without HEPES or NaCO_3_ but supplemented with glucose (25mM), pyruvate (1 mM), L-glutamine (1 mM), 4% Penicillin/streptomycin (5 U/ml) and 4% cefotaxim (100 µg/ml) (ACS Dobfar Generics S.A., Luxembourg, Belgium), pH 7.6). Cells were incubated in a CO_2_ free incubator at 37^o^C for 1 hour to allow temperature and pH equilibration where after the microplate with cells was loaded into the XF24 and the oxygen consumption rate of each well was measured over a period of 100 minutes. The drugs oligomycin (0.5 µM), FCCP (0.3 µM) and antimycin A (2.0 µM) were in turn added to each well. Oligomycin inhibits the mitochondrial ATP synthase and addition is correlated with a drop in oxygen consumption corresponding to O_2_ used for oxidative phosphorylation of ATP (ATP turnover. See fig 2). FCCP uncouples the mitochondrial membrane potential and addition is correlated with an increase in O_2_ consumption corresponding to maximal respiratory capacity. Antimycin A inhibits cytochrome c reductase that disrupts the formation of the proton gradient across the mitochondrial inner membrane. Addition results in a decrease in oxygen consumption corresponding to maximal amount of O_2_ that can be used for mitochondrial respiration (Respiratory capacity. See figure 2). The oxygen consumption measured after antimycin A corresponds to the oxygen used by other cellular processes than the electron transport chain (oxphos-independent oxygen consumption. See figure 2).
