# Supplementary material for: Enterococcus faecalis Infection Causes Inflammation, Intracellular Oxphos-Independent ROS Production, and DNA Damage in Human Gastric Cancer Cells
Source: PLoS One. 2013 Apr 30;8(4):e63147. doi: 10.1371/journal.pone.0063147 (PMC3639970; doi:10.1371/journal.pone.0063147)
Supplement: File S2 — (DOCX) [file pone.0063147.s007.docx]

#### File S2

If more than one probe set on the microarrays mapped to the same gene symbol then the max intensity value was used as representative gene expression level. Only gene sets of size 5 to 1000 were considered. GAGE^27^ gene set score (two-sample t-statistic) was calculated for each gene set as a measure of the enrichment in regulatory activity of genes in the corresponding pathway compared to a background of random gene sets of the same size constructed from all genes. For each experimental condition, log_2_-fold changes were calculated for all genes between each combination of treatment and control microarray (1-on-1 GAGE scheme) and used as gene-level score in the gene set statistic. For gene sets with induction/repression annotation (up/down regulation) the t-statistic used by GAGE was modified to improve statistical power by taking advantage of the directional information.

For a pair of gene sets with up/down information (name suffix ‘_UP’/‘_DN’), we merged the paired gene sets and modified GAGE as follows. Gene set mean, $m_{t}$, and standard deviation, $s_{t}$, was based on *transformed* log_2_-fold values by switching the sign for those genes in the gene set that ought to be down regulated by the activated pathway. GAGE parameterizes random gene sets by the mean, $M$, and standard deviation, $S$, of the background of log_2_-fold changes for all $N$ measured genes. For a pair of gene sets of total size $n$ composed of $n_{u}$ up and $n_{d}$ down regulated genes we replaced $M$ and $S$ with the transformed values

$$M_{t}=\frac{n_{u}M-n_{d}M}{n} \text{and}\text{ } S_{t}=\frac{1}{n(N-1)}\left( n_{u}\sum_{i=1}^{N} \left( f_{i}-M_{t} \right)^{2}+n_{d}\sum_{i=1}^{N} \left( -f_{i}-M_{t} \right)^{2} \right)$$

where $f_{i}$ is the raw log_2_-fold change for gene number $i$. The transformed parameters are plugged into the expression for the original t-statistic yielding the enrichment score

$$t=\left( m_{t}-M_{t} \right)/{\sqrt{\left( {{s_{t}}^{2}}/{n+{S_{t}^{2}}/n} \right)}}.$$

The original software implementation (R) of GAGE was modified accordingly to perform these calculations.

For gene sets of canonical pathways, gene set and background means and standard deviations were calculated based on $|f_{i}|$ as described by Luo et al to reflect that for individual genes in activated pathways the direction of gene regulation is in general unknown.
